# On predictive inference for intractable models via approximate Bayesian computation

**DOI:** 10.1007/s11222-022-10163-6

**Published:** 2023-02-09

**Authors:** Marko Järvenpää, Jukka Corander

**Affiliations:** 1 Department of Biostatistics, University of Oslo, Oslo, Norway; 2 Department of Mathematics and Statistics, Helsinki Institute of Information Technology (HIIT), University of Helsinki, Helsinki, Finland; 3 Wellcome Sanger Institute, Hinxton, Cambridgeshire, UK

**Keywords:** Approximate Bayesian computation, Posterior predictive distribution, Selection of summary statistics, Predictive sufficiency, Intractable dynamic models

## Abstract

Approximate Bayesian computation (ABC) is commonly used for parameter estimation and model comparison for intractable simulator-based statistical models whose likelihood function cannot be evaluated. In this paper we instead investigate the feasibility of ABC as a generic approximate method for predictive inference, in particular, for computing the posterior predictive distribution of future observations or missing data of interest. We consider three complementary ABC approaches for this goal, each based on different assumptions regarding which predictive density of the intractable model can be sampled from. The case where only simulation from the joint density of the observed and future data given the model parameters can be used for inference is given particular attention and it is shown that the ideal summary statistic in this setting is minimal predictive sufficient instead of merely minimal sufficient (in the ordinary sense). An ABC prediction approach that takes advantage of a certain latent variable representation is also investigated. We additionally show how common ABC sampling algorithms can be used in the predictive settings considered. Our main results are first illustrated by using simple time-series models that facilitate analytical treatment, and later by using two common intractable dynamic models.

**Supplementary Information:**

The online version contains supplementary material available at 10.1007/s11222-022-10163-6.

## Introduction

Approximate Bayesian computation has become an important technique for statistical inference in various application fields such as epidemiology and genomics (McKinley et al. 2009; Numminen et al. 2013; Kypraios et al. 2017; Järvenpää et al. 2019), systems biology (Wilkinson 2019; Warne et al. 2019) and computational finance (Calvet and Czellar 2014; Frazier et al. 2019) to name just a few. ABC allows to approximate the posterior distribution of unknown model parameters and the posterior probabilities of models using only forward model simulations. It hence facilitates Bayesian inference in an intuitive—though usually only in an approximate and computationally intensive—manner when the likelihood function is intractable in the sense that its analytical form is unavailable, too complex to analyze or too expensive to evaluate. Sometimes the terminology of likelihood-free inference (LFI) or simulation-based inference is used for computational methods in this space.

Despite substantive amount of research on ABC methodology and applications (see e.g. Marin et al. 2012; Sisson et al. 2019; Pesonen et al. 2022) and the fundamental importance of *prediction* in statistical inference, the use of ABC in the context of *predictive inference* has not been widely studied. In this paper we investigate ABC as a generic technique to approximate the posterior predictive distribution of some future observations or missing data. We denote the unobserved (future or missing) data of interest by $\tilde{y}$, the observed data by *y* and the unknown model parameters by $\theta$. Throughout this paper we assume that the analytical forms of the likelihood function $\pi (\tilde{y},y\,|\,\theta )$ (and $\pi (y\,|\,\theta )$) and the conditional predictive density $\pi (\tilde{y}\,|\,y,\theta )$ are intractable. We especially study a rather general situation where one can jointly simulate from $\pi (\tilde{y},y\,|\,\theta )$ for each $\theta$ but, importantly, not necessarily from $\pi (\tilde{y}\,|\,y,\theta )$. No specific assumptions of the model structure are made but we also separately analyze such models that facilitate simulation from $\pi (\tilde{y}\,|\,v,y,\theta )$ where *v* are unobserved (finite-dimensional) latent variables. The goal is to infer the unobserved data $\tilde{y}$ and possibly also $\theta$ given the observations *y*. We consider only an offline setting where data are not obtained sequentially and where summarized data is used as common also in more conventional ABC inference tasks.

An earlier approach for ABC prediction, used in applied work e.g. by McKinley et al. (2009), Canale and Ruggiero (2016), Pesonen et al. (2022) and studied theoretically in the context of financial time-series models that facilitate partial analytical treatment by Frazier et al. (2019), consists of first using ABC to approximate $\pi (\theta \,|\,y)$ and then simulating directly from $\pi (\tilde{y}\,|\,y,\theta )$. However, the latter step can be infeasible or its implementation may require laborious model-specific derivations. In contrary, sampling from $\pi (\tilde{y},y\,|\,\theta )$, in particular, can often be carried out easily—typically using the same algorithm as sampling from $\pi (y\,|\,\theta )$ which is in any case needed for ABC. The main advantage of the proposed ABC methods is that they allow predictive inference very generally—even when the model is specified as a complex computer code that jointly outputs pseudo-data from $\pi (\tilde{y},y\,|\,\theta )$. An example would be a temporal Markov model defined via an intractable transition density. If e.g. only some elements of the last state $y_{\tau }$ were observed, then forward simulation of future data $\tilde{y}$ directly via $\pi (\tilde{y}\,|\,y,\theta ) = \pi (\tilde{y}\,|\,y_{\tau },\theta )$ becomes infeasible. Applications include infectious diseases modelling and stochastic biochemical networks, among others, where (semi-)Markov and stochastic differential equation models are used (see e.g. Picchini 2014; Kypraios et al. 2017; Tancredi 2019; Wilkinson 2019; Buckwar et al. 2020) and where incomplete measurements are often only available. See also Pesonen et al. (2022) where realistic intractable dynamic models are used for prediction, though in a simpler setting as considered here. The focus of this paper is, however, on understanding the predictive ABC approximations and not on particular applications. We also offer some new insight on the aforementioned earlier ABC prediction approach and argue that, if applicable, it can be expected to produce more accurate approximation of the posterior predictive distribution than the more generic methods we propose.

Predictive ABC methods are also useful for parameter estimation and model comparison of intractable models. For example, computation of the posterior predictive distribution for the hypothetical future data to be observed given the candidate experimental design and current data is required in sequential Bayesian experimental design. This requires ABC resolution when the model is intractable, see Hainy et al. (2016), Kleinegesse et al. (2021). Also, various methods for model assessment and comparison based on data splitting (see e.g. Vehtari and Ojanen 2012; Bürkner et al. 2020), which are yet to be extended to the ABC setting, require the ability to predict data in a hold-out set (or in a cross-validation fold) given the rest of the data. Predictive inference with tractable models may sometimes require ABC: Bayesian inference could be based on summarized data to make the likelihood function better behaving (see e.g. Wood 2010; Fasiolo et al. 2016) or to alleviate the effects of possible outlier observations and model misspecification (see e.g. Lewis et al. (2021)) which can render inference intractable. These potential applications are however not the focus of this work.

In this paper we only analyze generic ABC prediction methods which are widely applicable, fairly simple to implement and likely easy for a practitioner to understand. It is however worth emphasizing that, obviously, taking advantage of the model structure at hand or its partial analytical tractability, whenever feasible, might lead to more accurate or efficient model-specific algorithms. An example would be hidden Markov and state-space models (SSM) with intractable observation models which have been studied by Jasra et al. (2012), Martin et al. (2014), Calvet and Czellar (2014), Jasra (2015), Vankov et al. (2019). If ABC inference can be based on the full data (instead of summary statistics) as assumed in these works, and if the unobserved latent states are also to be estimated, the model-specific ABC algorithms in the above works are presumably most suitable if extended for the prediction task. Similar remark holds for Markov jump processes with tractable noise models that in some settings facilitate asymptotically exact particle MCMC methods, see e.g. Golightly and Wilkinson (2011), Wilkinson (2019), Warne et al. (2020). We also consider some SMMs but this is mainly for the simplicity of demonstration. See also Martin et al. (2019) where summary statistics based on auxiliary models are used in ABC parameter estimation with SMMs.

The rest of this paper is organized as follows: In Sect. 2 we briefly review the key ideas of Bayesian predictive inference and ABC. In Sect. 3 we first discuss the aforementioned common approach for predictive ABC. We then develop the new ABC prediction approaches and analyze some of their properties. We especially study the selection of summary statistics and observe that these ideally need to be minimal predictive sufficient instead of merely minimal sufficient (in the ordinary sense). In the alternative approach where an assumption of latent variable representation is made, a statistic that is jointly sufficient for both the parameters $\theta$ and the latent variables *v* relevant for the prediction task is appropriate. After that, in Sect. 4 we show how common ABC samplers such as ABC-MCMC (Marjoram et al. 2003) and PMC-ABC (Beaumont et al. 2009) can be used for predictive inference and discuss some practical aspects relevant for applications. Section 5 presents numerical experiments with M/G/1 queue and Lotka–Volterra models which illustrate the main theoretical findings and the quality of the predictive ABC approximations. Section 6 summarizes the main results and suggests topics for future work. Additional analysis and technical details can be found in the Supplementary material.

## Background

We denote the observed data as $y=y_{1:n}=(y_1,\ldots ,y_n)$ and the unobserved data of interest as $\tilde{y}=(\tilde{y}_1,\ldots ,\tilde{y}_{\tilde{n}})$. We assume a parametric model $\pi (\tilde{y},y\,|\,\theta ) = \pi (\tilde{y}\,|\,y,\theta )\pi (y\,|\,\theta )$ with a tractable prior density $\pi (\theta )$ for the unknown model parameters $\theta \in \Theta \subset \mathbb {R}^{p}$. Unless explicitly stated otherwise, possible latent variables, which we typically denote by *v* (and $\tilde{v}$), are not included to $\theta$ in our notation. The individual data points $y_i\in \mathscr {Y}_i\subset \mathbb {R}^{d_i}$ are typically neither independent nor identically distributed and are associated with known input variables $x_i\in \mathscr {X}_i$ with $x=x_{1:n}=(x_1,\ldots ,x_n)$. The same holds for unobserved data $\tilde{y}$ with inputs $\tilde{x}=(\tilde{x}_1,\ldots ,\tilde{x}_{\tilde{n}})$. In the case of a time-series model, each $x_i$ would typically denote time and $y_i$ related observation. We however suppress the dependence on *x* and $\tilde{x}$ for brevity.

### Predictive inference

Inference about the unobserved (future) data $\tilde{y}$ after observing *y*, which is specifically called predictive inference, is in Bayesian statistics done using the posterior distribution

1 $$\begin{aligned} \pi (\tilde{y}\,|\,y) = \int \pi (\tilde{y},\theta \,|\,y) \mathop {}\!\textrm{d}\theta = \int \pi (\tilde{y}\,|\,\theta ,y) \pi (\theta \,|\,y) \mathop {}\!\textrm{d}\theta . \end{aligned}$$

This quantity is called the posterior predictive distribution. It captures the uncertainty in $\tilde{y}$ both due to the stochastic model and the incomplete knowledge of the model parameters $\theta$. The latter is represented by the posterior distribution

2 $$\begin{aligned} {\pi (\theta \,|\,y) = \frac{\pi (y\,|\,\theta )\pi (\theta )}{\int \pi (y\,|\,\theta ) \pi (\theta ) \mathop {}\!\textrm{d}\theta } \propto \pi (y\,|\,\theta ) \pi (\theta ),} \end{aligned}$$

where $\pi (y)=\int \pi (y\,|\,\theta ) \pi (\theta ) \mathop {}\!\textrm{d}\theta$ is the marginal likelihood. As mentioned in O’Hagan and Forster (2004, Section 3), $\theta$ can be seen as a nuisance parameter in predictive inference. However, both $\tilde{y}$ and $\theta$ might be of interest in some applications.

Although we are mainly concerned with prediction in this paper, (1) and our proposed ABC methods similarly apply when $\tilde{y}$ denotes missing observations to be estimated. Furthermore, in some applications latent variables $\tilde{v}$ are of the main interest instead of the related future data $\tilde{y}$. For example, consider a SSM, where $v=v_{1:n}$ denote the unobserved latent states and $y=y_{1:n}$ the corresponding observations and where the transition and measurement distributions are

3 $$\begin{aligned} \pi (v_i\,|\,v_{i-1},\theta ), \quad \pi (y_i\,|\,v_i,\theta ), \quad i=1,\ldots ,n, \end{aligned}$$

respectively. The posterior predictive for e.g. $\tilde{v}=v_{n+1}$ is still obtained by applying (1) when $\tilde{y}$ is replaced by $(\tilde{v},\tilde{y})$ and *v* is included to $\theta$ (or marginalized). Hence, we do not handle cases like this separately in what follows. When possible, taking the latent variable structure such as (3) into account can be useful for computational reasons as argued by Jasra (2015) and as considered in Sect. 3.4 of this paper.

The posterior predictive distribution (1) typically needs to be computed numerically. A common approach is to use the estimator

4 $$\begin{aligned} \pi (\tilde{y}\,|\,y) \approx \widehat{\pi }(\tilde{y}\,|\,y)~ {:}{=}~ \frac{1}{m}\sum _{j=1}^m \pi (\tilde{y}\,|\,\theta ^{(j)},y), \end{aligned}$$

where $\theta ^{(j)}, j=1,\ldots ,m$, are (possibly correlated) samples from the posterior $\pi (\theta \,|\,y)$ obtained e.g. by some Markov chain Monte Carlo (MCMC) method. If evaluating the conditional predictive density $\pi (\tilde{y}\,|\,\theta ,y)$ needed for (4) is difficult, samples from $\pi (\tilde{y}\,|\,y)$ can be obtained by simulating $\tilde{y}^{(j)}$ from $\pi (\tilde{y}\,|\,\theta ^{(j)},y)$ for $j=1,\ldots ,m$. Theoretical properties of the resulting approximations have recently been extensively studied by Krüger et al. (2021). All in all, provided that one can either evaluate the conditional predictive density or sample from it efficiently, no additional computational challenges arise in principle.

### Approximate Bayesian computation

Before we study predictive inference for intractable models using ABC, we briefly review standard ABC parameter inference. By this we mean the task of approximating the posterior distribution $\pi (\theta \,|\,y)$ using only forward model simulations from $\pi (y\,|\,\theta )$. More detailed overviews of ABC than given here can be found in Marin et al. (2012), Lintusaari et al. (2017), Sisson et al. (2019). The basic ABC rejection sampler (Tavaré et al. 1997; Pritchard et al. 1999) consists of repeating the following steps for $i=1,\ldots ,m$:

1. Simulate $\theta ^{(i)}$ from the prior $\pi (\theta )$,
2. Simulate pseudo-data $z^{(i)}$ from the model given $\theta ^{(i)}$,
3. Accept $$\theta ^{(i)}$$ if $$\Delta (s(y),s(z^{(i)}))\le h$$.

The summary statistic^1^$s:\prod _{i}\mathscr {Y}_i \rightarrow \mathbb {R}^d$ in A3 is used to summarize the potentially high-dimensional observed data *y* and the corresponding simulated data sets $z^{(i)}$. The discrepancy function $\Delta :\mathbb {R}^d\times \mathbb {R}^d\rightarrow \mathbb {R}_{+}$ and the threshold parameter $h\ge 0$ are further used to compare the similarity of the summary statistics. Commonly $\Delta$ is some (weighted) norm $\Vert \cdot \Vert$ in $\mathbb {R}^d$, that is, $\Delta (s_y,s_z)=\Vert s_y-s_z\Vert$, but other choices are also possible. We often denote the observed summary statistic using a short-hand notation $s_y~{:}{=}~s(y)$ and similarly $s_z~{:}{=}~s(z)$. The accepted samples in A3 are considered as independent draws from the approximate posterior distribution called the ABC posterior.

The ABC rejection sampler outlined above and the more advanced samplers discussed in the context of ABC prediction in Sect. 4 target the ABC posterior

5 $$\begin{aligned}{} & {} \widehat{\pi }_{h}(\theta \,|\,s_y) ~{:}{=}~ \frac{\int K_h(\Delta (s_y,s_z)) \pi (s_z\,|\,\theta ) \pi (\theta ) \mathop {}\!\textrm{d}s_z}{\iint K_h(\Delta (s_y,s_z)) \pi (s_z\,|\,\theta ) \pi (\theta ) \mathop {}\!\textrm{d}s_z \mathop {}\!\textrm{d}\theta }\nonumber \\{} & {} \quad \propto \int K_h(\Delta (s_y,s_z)) \pi (s_z\,|\,\theta ) \pi (\theta ) \mathop {}\!\textrm{d}s_z, \end{aligned}$$

where $K_h(r) \propto \mathbb {1}_{r\le h}$. Other choices of the kernel $K_h:\mathbb {R}_{+}\rightarrow \mathbb {R}_{+}$, such as the Gaussian kernel $K_h(r) \propto \exp (-r^2/(2h^2))$ where $h> 0$ is a bandwidth parameter, are also possible. The intractable likelihood $\pi (y\,|\,\theta )$ in (2) has essentially been replaced by the ABC likelihood $\widehat{\pi }_{h}(s_y\,|\,\theta ) ~{:}{=}~ \int K_h(\Delta (s_y,s_z)) \pi (s_z\,|\,\theta ) \mathop {}\!\textrm{d}s_z$ to obtain (5). The quality of this approximation depends on the selection of the summary statistic *s* and the threshold (bandwidth) parameter *h* in particular. In principle *s* can be the identity mapping which produces no loss of information but, unless the data is low-dimensional, data summarization is necessary for computational efficiency. Similarly, the threshold *h* needs to be selected to balance the tradeoff between approximation accuracy and computational efficiency.

It is possible to define ABC posterior also via multiple discrepancies $\Delta ^{(j)}(s^{(j)}(y),s^{(j)}(z))$, each based on different summary statistic $s^{(j)}$ and threshold $h^{(j)}$, by replacing the kernel $K_h(\Delta (s_y,s_z))$ in (5) with a product of kernels $\prod _j K_{h^{(j)}}\left[ {\Delta ^{(j)}(s^{(j)}(y),s^{(j)}(z))}\right]$. Such an approach with uniform kernels $K_{h^{(j)}}$ has been used by Pritchard et al. (1999), Ratmann et al. (2009), Numminen et al. (2013) for standard ABC parameter inference. The resulting alternative ABC posterior approximation differs from the more common one in that it defines a different acceptance region in the summary statistic space. Wilkinson (2013) has shown that these approaches can be interpreted as different measurement error models.

## Predictive inference using ABC

In Sect. 3.1 we first consider the situation with tractable $\pi (\tilde{y}\,|\,y,\theta )$ which typically does not pose significant additional computational or statistical challenges over standard ABC inference. We then analyze a more challenging situation where only joint simulation from $\pi (\tilde{y},y\,|\,\theta )$ is used (Sect. 3.2). Some special circumstances relevant for all methods are considered in Sect. 3.3 and, finally, taking advantage of a latent variable representation is studied in Sect. 3.4. Table 1 shows a summary of the ABC prediction methods.

**Table 1:** A simplified overview of the ABC prediction methods considered in this paper. An alternative to ABC-P is also briefly analyzed in Supplementary material A. Numerical methods are considered in Sect. 4

Method	Approximation	Sampling feasible from	(Ideal) Summary statistic	Details
ABC-F	$\widehat{\pi }^{\,\smash {\text {(F)}}}_{h}(\tilde{y}\,|\,y; s_y)$, (6)	$\pi (\tilde{y}\,|\,\theta ,y)$ and $\pi (y\,|\,\theta )$	Parametric sufficient (for $\theta$)	Sect. 3.1
ABC-P	$\widehat{\pi }^{\,\smash {\text {(P)}}}_{h}(\tilde{y} \,|\,s_y)$, (8)	$\pi (\tilde{y},y\,|\,\theta )$ (jointly)	Predictive sufficient (for $\tilde{y}$)	Sect. 3.2
ABC-L	$\widehat{\pi }^{\,\smash {\text {(L)}}}_h(\tilde{y}\,|\,y;s_y)$, (26)	$\pi (\tilde{y}\,|\,y,v,\theta )$ and $\pi (y,v\,|\,\theta )$	Parametric sufficient (for $\theta$,*v*)	Sect. 3.4

### Tractable simulation from conditional predictive density

When the exact posterior $\pi (\theta \,|\,y)$ in (1) is replaced by the ABC posterior^2^$\widehat{\pi }_h(\theta \,|\,s_y)$, the following approximation for the posterior predictive distribution $\pi (\tilde{y}\,|\,y)$ results:

6 $$\begin{aligned} \pi (\tilde{y}\,|\,y) \approx \widehat{\pi }^{\,\smash {\text {(F)}}}_{h}(\tilde{y}\,|\,y; s_y) ~{:}{=}~ \int \pi (\tilde{y}\,|\,\theta ,y) \widehat{\pi }_h(\theta \,|\,s_y) \mathop {}\!\textrm{d}\theta . \end{aligned}$$

This intuitive method has been used for applied work by McKinley et al. (2009), Canale and Ruggiero (2016), Pesonen et al. (2022) but without detailed analysis. Frazier et al. (2019) analyzed this approach theoretically and showed that under certain technical conditions the approximate posterior predictive in a sense coincidences with the exact one as the data size becomes arbitrarily large and as $h\rightarrow 0$. Their numerical results interestingly suggest that the approximate posterior predictive can be accurate even if the corresponding ABC posterior of $\theta$ provides a poor approximation.

In a typical LFI scenario one cannot evaluate $\pi (\tilde{y}\,|\,y,\theta )$. Samples from $\widehat{\pi }^{\,\smash {\text {(F)}}}_{h}(\tilde{y}\,|\,y; s_y)$ in (6) can be obtained using a similar two stage approach as in Sect. 2.1: One first draws $\theta ^{(i)} \sim \widehat{\pi }_h(\theta \,|\,s_y)$ using some standard ABC sampler and finally draws $\tilde{y}^{(i)} \sim \pi (\tilde{y}\,|\,\theta ^{(i)},y)$. We use the abbreviation ABC-F for this approach where “F”^3^ informs that this method is directly based on *forward simulation* via $\pi (\tilde{y}\,|\,y,\theta )$. The ABC parameter draws can be recycled for other prediction tasks involving the same model and observed data *y*.

A limitation of ABC-F, mentioned already in Sect. 1, is that sampling from $\pi (\tilde{y}\,|\,\theta ,y)$ may be infeasible when the model is (truly) intractable. Frazier et al. (2019) used ABC-F to improve computational efficiency of certain time-series models which facilitate partial analytical treatment and access to $\pi (\tilde{y}\,|\,\theta ,y)$. Also, simulation from $\pi (\tilde{y}\,|\,\theta ,y)$ may be feasible when the model is intractable but is Markovian whose last measured state is fully observed. An important special case where ABC-F applies is when $\pi (\tilde{y}\,|\,y,\theta ) = \pi (\tilde{y}\,|\,\theta )$. In particular, the conditional predictive distribution of a model that produces i.i.d. data has the same form as the corresponding likelihood function $\pi (y\,|\,\theta )$ so that it is in principle possible to even recycle samples generated from $\pi (y\,|\,\theta )$ during the ABC sampling from $\widehat{\pi }_h(\theta \,|\,s_y)$. A special instance of this is the standard posterior predictive checking of Gelman et al. (1996, 2013, Section 6.3). In this method *independent* replicated data sets are drawn from the posterior predictive which are then compared against the observed data *y*. This can be implemented using only repeated sampling from $\pi (y\,|\,\theta )$ in the ABC case.

### Intractable simulation from conditional predictive density

We now consider the situation where we do not necessary have access to $\pi (\tilde{y}\,|\,\theta ,y)$ but joint simulation from $\pi (\tilde{y},y\,|\,\theta )$ is feasible. We first write (1) as

7 $$\begin{aligned} \pi (\tilde{y}\,|\,y) = \frac{\pi (\tilde{y}, y)}{\pi (y)} = \frac{\int \pi (\tilde{y}, y\,|\,\theta ) \pi (\theta ) \mathop {}\!\textrm{d}\theta }{\iint \pi (\tilde{y}, y\,|\,\theta ) \pi (\theta ) \mathop {}\!\textrm{d}\theta \mathop {}\!\textrm{d}\tilde{y}}. \end{aligned}$$

If we then replace $\pi (\tilde{y}, y\,|\,\theta )$ in (7) with the quantity $\widehat{\pi }_{h}(\tilde{y},s_y\,|\,\theta ) ~{:}{=}~ \int K_h(\Delta (s_y,s_z)) \pi (\tilde{y},s_z\,|\,\theta ) \mathop {}\!\textrm{d}s_z$, in a similar manner as $\pi (y\,|\,\theta )$ is replaced by $\widehat{\pi }_{h}(s_y\,|\,\theta )$ to obtain (5), the following approximation for the posterior predictive results:

8 $$\begin{aligned} \begin{aligned}&\pi (\tilde{y}\,|\,y) \approx \widehat{\pi }^{\,\smash {\text {(P)}}}_{h}(\tilde{y} \,|\,s_y)\\&~{:}{=}~ \frac{\iint K_h({\Delta (s_y,s_z)}) \pi (\tilde{y}, s_z\,|\,\theta ) \pi (\theta ) \mathop {}\!\textrm{d}s_z \mathop {}\!\textrm{d}\theta }{\iiint K_h({\Delta (s_y,s_z)}) \pi (\tilde{y}, s_z\,|\,\theta ) \pi (\theta ) \mathop {}\!\textrm{d}s_z \mathop {}\!\textrm{d}\theta \mathop {}\!\textrm{d}\tilde{y}} \\&\propto \iint K_h({\Delta (s_y,s_z)}) \pi (\tilde{y}, s_z\,|\,\theta ) \pi (\theta ) \mathop {}\!\textrm{d}s_z \mathop {}\!\textrm{d}\theta . \end{aligned} \end{aligned}$$

We similarly obtain the following joint ABC posterior distribution

9 $$\begin{aligned}{} & {} \pi (\tilde{y},\theta \,|\,y) \approx \widehat{\pi }^{\,\smash {\text {(P)}}}_{h}(\tilde{y},\theta \,|\,s_y) \nonumber \\{} & {} \quad \propto \int K_h({\Delta (s_y,s_z)}) \pi (\tilde{y}, s_z\,|\,\theta ) \pi (\theta ) \mathop {}\!\textrm{d}s_z. \end{aligned}$$

These approximations depend on the simulator-based model only via $\pi (\tilde{y}, s_z\,|\,\theta )$ which is the joint distribution of the future (pseudo-)data $\tilde{y}$ and the summarized pseudo-data $s_z$ given parameter $\theta$. Obviously, we can simulate from this density by first using the simulator-based model to draw from $\pi (\tilde{y},z\,|\,\theta )$ and then applying the mapping $(\tilde{y},z)\mapsto (\tilde{y},s(z))$.

The rest of this section is devoted to the analysis of the approximations (8) and (9) which we refer as ABC-P. A related alternative, based on a “nested” ABC approximation of $\pi (\tilde{y}\,|\,\theta ,y)$ in the ABC-F approach, is analyzed in Supplementary material A. We first observe that marginalizing $\theta$ in (9) naturally produces (8) while marginalizing $\tilde{y}$ reasonably results the standard ABC posterior approximation $\widehat{\pi }_{h}(\theta \,|\,s_y)$. We also notice the following connection between ABC-P and ABC-F: If we use the basic fact $\pi (\tilde{y}, s_z\,|\,\theta ) = \pi (\tilde{y}\,|\,s_z, \theta ) \pi (s_z\,|\,\theta )$ in (8) and then replace $\pi (\tilde{y}\,|\,s_z, \theta )$ with $\pi (\tilde{y}\,|\,s_y, \theta )$ in the resulting formula (which is a reasonable approximation in that the integrand of (8) is approximately zero when *h* is small and when $s_y$ is substantially different from $s_z$), we obtain a special case $\widehat{\pi }^{\,\smash {\text {(F)}}}_{h}(\tilde{y}\,|\,s_y; s_y)$ of the ABC-F approximation. Importantly, this connection additionally implies that the ABC-F and ABC-P target densities coincide in the case of independent data because then we have $\pi (\tilde{y}\,|\,s_z, \theta ) = \pi (\tilde{y}\,|\,\theta )$.

#### Relation to exact posterior predictive distribution

We analyze the ABC-P target posterior predictive based on the observed summary $s_y$ when the uniform kernel $K_h(r) \propto \mathbb {1}_{r\le h}$ is used. We adopt the setting of ABC samplers (Sect. 4.1) where $h_t$ is a sequence of decreasing thresholds. Lebesgue measure is here denoted as $|\cdot |$. We make the following assumptions:

1. The random vector $(\tilde{y},\theta ,s_y)$ has a joint density function $\pi (\tilde{y},\theta ,s_y)$ with respect to Lebesgue measure,
2. The acceptance region $\mathcal {A}_t~{:}{=}~\{u\in \mathbb {R}^d:\Delta _t(u,s_y)\le h_t\}$ (or $\mathcal {A}_t~{:}{=}~\{u\in \mathbb {R}^d:\Delta ^{(j)}_t(u,s_y)\le h^{(j)}_t \,\forall j\}$ if multiple discrepancies are used) is Lebesgue measurable for each *t*,
3. $$\pi (s_y)>0$$,
4. $$\lim _{t\rightarrow \infty }|\mathcal {A}_t|=0$$,
5. Sets $\mathcal {A}_t$ have *bounded eccentricity*, that is, for each $\mathcal {A}_t$ there exists a ball $\mathcal {B}_t=\{u\in \mathbb {R}^d:\Vert u-s_y\Vert \le r_t\}$ such that $\mathcal {A}_t\subset \mathcal {B}_t$ and $|\mathcal {A}_t|\ge c|\mathcal {B}_t|$ with some constant $c>0$.

These assumptions are similar to those used by Prangle (2017), who analyzed the convergence of the ABC posterior of $\theta$, except that we need to consider future data $\tilde{y}$ in (C1). Also, we allow multiple discrepancies in (C2) which is relevant for an approach in Sect. 4.2. The discrepancy $\Delta _t$ in (C2) may depend on *t* although this dependence plays no important role in this paper. We refer to Prangle (2017, Section 2.3) for further discussion on the assumptions which also applies to our predictive ABC setting.

The following result shows that the ABC-P target posterior predictive becomes arbitrarily accurate if the size of the acceptance region shrinks to zero appropriately which is ensured by the above assumptions. The proof of this result is given in Supplementary material B.1. It is easy to verify that (C2), (C4) and (C5) hold in a typical case where $\Delta (s_z,s_y)$ is some norm and the sequence $h_t$ is such that $h_t\rightarrow 0$ as $t\rightarrow \infty$.

##### Proposition 3.1

Assume that a uniform kernel is used and (C1–C5) are satisfied. Then, for almost all $(\tilde{y},\theta ,s_y)$ wrt. the joint density $\pi (\tilde{y},\theta ,s_y)$, it holds that

10 $$\begin{aligned}{} & {} \lim _{t\rightarrow \infty } \widehat{\pi }^{\,\smash {\text {(P)}}}_{h_t}(\tilde{y}\,|\,s_y) = \pi (\tilde{y}\,|\,s_y), \quad \nonumber \\{} & {} \lim _{t\rightarrow \infty } \widehat{\pi }^{\,\smash {\text {(P)}}}_{h_t}(\tilde{y},\theta \,|\,s_y) = \pi (\tilde{y},\theta \,|\,s_y). \end{aligned}$$

We additionally obtain $\widehat{\pi }^{\,\smash {\text {(P)}}}_{\infty }(\tilde{y}\,|\,s_y) = \pi (\tilde{y}) = \int \pi (\tilde{y}\,|\,\theta )\pi (\theta ) \mathop {}\!\textrm{d}\theta = \iint \pi (\tilde{y},y\,|\,\theta )\pi (\theta )\mathop {}\!\textrm{d}y\mathop {}\!\textrm{d}\theta$, that is, the ABC posterior predictive coincides with the prior predictive when $h=\infty$. A curiosity is that ABC-F leads to a different prior predictive density $\widehat{\pi }^{\,\smash {\text {(F)}}}_{\infty }(\tilde{y}\,|\,y; s_y)=\int \pi (\tilde{y}\,|\,\theta ,y)\pi (\theta )\mathop {}\!\textrm{d}\theta$ which however clearly agrees with $\widehat{\pi }^{\,\smash {\text {(P)}}}_{\infty }(\tilde{y}\,|\,s_y)$ if one formally sets $y=\varnothing$ in $\widehat{\pi }^{\,\smash {\text {(F)}}}_{\infty }(\tilde{y}\,|\,y; s_y)$. Using some results in Stein and Shakarchi (2005), Biau et al. (2015) one could likely extend Proposition 3.1 for more general kernel functions. See also Barber et al. (2015) for related analysis. In the following we however study the summary statistics selection as this analysis is more relevant for practice and differs from that of the standard ABC inference.

#### Selection of summary statistics

Recall that a statistic *s* is *(classical) sufficient* if the conditional density $\pi (y\,|\,s_y,\theta )$ does not depend on $\theta$ and *Bayes sufficient* if $\pi (\theta \,|\,y) = \pi (\theta \,|\,s_y)$ for each prior density $\pi (\theta )$ (and for almost all *y*). It can be shown that Bayes sufficiency is equivalent to classical sufficiency when $\theta$ is finite-dimensional, see e.g. Schervish (1995, Theorem 2.14). In what follows we hence consider only Bayes sufficiency^4^ which we call *parametric sufficiency* to surely distinguish it from other sufficiency conditions encountered below.

Under assumptions similar to our (C1)-(C5), the ABC posterior $\widehat{\pi }_{h_t}(\theta \,|\,s_y)$ converges to $\pi (\theta \,|\,s_y)$ as $t\rightarrow \infty$, see Prangle (2017) or (Sisson et al. (Sisson et al. 2019, Chapter 1.7) for a less precise analysis). Now, when the summary statistic *s* is parametric sufficient, the limiting ABC posterior $\pi (\theta \,|\,s_y)$ further agrees with the exact posterior $\pi (\theta \,|\,y)$. The ABC-F posterior predictive $\widehat{\pi }^{\,\smash {\text {(F)}}}_{h}(\tilde{y}\,|\,y; s_y)$ in (6) obviously depends on *h* and $s_y$ only via the ABC posterior $\widehat{\pi }_h(\theta \,|\,s_y)$ and hence agrees with the exact posterior predictive $\pi (\tilde{y}\,|\,y)$ in this scenario. (In Sect. 3.3 we nonetheless argue that parametric sufficiency is not a necessary condition for this result regarding ABC-F.) However, perhaps surprisingly, parametric sufficiency does not guarantee that an analogous result holds for either ABC-P limiting posterior predictive distributions in (10).

First, we define a summary statistic *s* to be *predictive sufficient* if

11 $$\begin{aligned} \pi (\tilde{y} \,|\,y) = \pi (\tilde{y} \,|\,s_y). \end{aligned}$$

If $\tilde{y}$ and *y* are conditionally independent given $\theta$ (in particular, if the data is i.i.d.) so that $\pi (\tilde{y} \,|\,y, \theta ) = \pi (\tilde{y} \,|\,\theta )$, then parametric sufficiency implies predictive sufficiency (11) because $\pi (\tilde{y} \,|\,y) = \int \pi (\tilde{y} \,|\,y,\theta )\pi (\theta \,|\,y)\mathop {}\!\textrm{d}\theta = \int \pi (\tilde{y} \,|\,\theta )\pi (\theta \,|\,s_y)\mathop {}\!\textrm{d}\theta = \pi (\tilde{y} \,|\,s_y)$. This result does not hold more generally as we see later in Example 3.1. Classical, Bayes and predictive sufficiency are however all equivalent in a setting of infinitely exchangeable observations of Bernardo and Smith (1994, Section 4.5) but this situation is not particularly relevant for ABC applications.

When the summary statistic *s* satisfies the predictive sufficiency condition (11), it is immediately seen that the ABC-P limiting predictive posterior approximation $\pi (\tilde{y}\,|\,s_y)$ of Proposition 3.1 coincides with the exact posterior predictive $\pi (\tilde{y}\,|\,y)$. The choice $s(y)=y$ would trivially satisfy this condition but the summary statistic should be low-dimensional for computational reasons as in standard ABC inference. We conclude that the summary statistic should ideally be minimal predictive sufficient in the sense of (11) to estimate $\tilde{y}$ by using ABC-P.

Another, perhaps less known, form of predictive sufficiency exists (Skibinsky 1967; Lauritzen 1974; Bjørnstad 1996). This *stronger form of predictive sufficiency* requires the following two conditions:

12 $$\begin{aligned} \pi (\theta \,|\,y)&= \pi (\theta \,|\,s_y), \,\text {(that is, } s \text { is parametric sufficient)}, \end{aligned}$$

13 $$\begin{aligned} \pi (\tilde{y} \,|\,y, \theta )&= \pi (\tilde{y} \,|\,s_y, \theta ). \end{aligned}$$

This definition is most natural for our ABC setting but other equivalent definitions exist, see Bjørnstad (1996, Section 3.1). If *s* is predictive sufficient in the sense of (12) and (13), then *s* is obviously parametric sufficient but the converse does not hold in general. If $\pi (\tilde{y} \,|\,\theta , y) = \pi (\tilde{y} \,|\,\theta )$, then (13) is satisfied and this definition of predictive sufficiency reverts to that of parametric sufficiency.

When (12) and (13) both hold, we have

14 $$\begin{aligned}&\pi (\tilde{y},\theta \,|\,y) = \pi (\tilde{y}\,|\,y,\theta )\pi (\theta \,|\,y) \nonumber \\&= \pi (\tilde{y}\,|\,s_y,\theta )\pi (\theta \,|\,s_y) = \pi (\tilde{y},\theta \,|\,s_y). \end{aligned}$$

Predictive sufficiency in the sense of (12) and (13) thus implies predictive sufficiency in the sense of (11). This offers a more intuitive way of finding summary statistics for ABC-P as compared to (11): One can take some parametric sufficient statistic and complement it with additional statistics that satisfy (13). This approach may however not produce minimal predictive sufficient statistic in the sense of (11). In practice, where low-dimensional sufficient statistics may not exist and are in any case difficult to find due to the intractability of the model, one can take some “usual” summary statistic deemed informative for $\theta$ and try to device additional ones that could be informative for prediction in the sense of (13).

When the summary statistic $s$ satisfies (14), the joint ABC-P limiting approximation $\pi (\tilde{y},\theta \,|\,s_y)$ of Proposition 3.1, clearly coincides with $\pi (\tilde{y},\theta \,|\,y)$. This result is relevant when both $\tilde{y}$ and $\theta$ are of interest. Of course, $s$ should be low-dimensional also in this case.

##### Example 3.1

(Markov model) This example illustrates the effect of the summary statistics and threshold on the ABC posterior predictive. Consider a discrete-time process

15 $$\begin{aligned} y_t\,|\,y_{t-1},\theta \sim {{\,\mathrm{\mathcal {N}}\,}}(c+\phi y_{t-1},\sigma ^2), \end{aligned}$$

where $t=1,2,\ldots ,n$ with $n>1$ and $y_0=0$. The parameters are $\theta =(c,\phi ,\sigma ^2)\in \mathbb {R}\times (-1,1)\times \mathbb {R}_{+}$ though $\phi$ and $\sigma ^2$ are here considered known. Consider first the task of estimating *c* when $y=y_{1:n}=(y_1,\ldots ,y_n)\in \mathbb {R}^n$ is directly observed and the prior for *c* is $\pi (c)\propto 1$. Then the weighted average $\bar{y}_{\phi }$ shown below in (16) is a parametric sufficient statistic and when a Gaussian kernel ${{\,\mathrm{\mathcal {N}}\,}}(\bar{y}_{\phi }\,|\,\bar{z}_{\phi }, h^2)$ is used, we have

16 $$\begin{aligned} \widehat{\pi }_h(c\,|\,\bar{y}_{\phi }) = {{\,\mathrm{\mathcal {N}}\,}}(c\,|\,\bar{y}_{\phi },\sigma ^2/n + h^2),~~ \bar{y}_{\phi }~{:}{=}~ \frac{(1-\phi )\sum _{i=1}^{n-1}y_i + y_n}{n}. \nonumber \\ \end{aligned}$$

The exact posterior $\pi (c\,|\,y)$ is obtained by setting $h=0$ in (16). The derivation of (16) and the other results to follow are outlined in Supplementary material B.2.

We next study ABC-P and ABC-F posterior predictive densities of $\tilde{y}=y_{n+1}$. When the Gaussian kernel is again used between the summary statistics under consideration, we obtain^5^17181920While the statistic $\bar{y}_{\phi }$ is sufficient for estimating *c*, (18) shows that it is neither sufficient for $y_{n+1}$ nor a good choice in practice. Namely, even when $h=0$, the mean prediction of (18) can be very different than that of the exact posterior predictive in (17). Furthermore, it follows from (19) that the variance in (18) is always larger than that of (17) except for some special cases such as the i.i.d. data case $\phi =0$ where they are equal. On the other hand, the predictive ABC-P posteriors based on $(\bar{y}_{\phi },y_n)$ and $\mathring{y}_{\phi }~{:}{=}~ \bar{y}_{\phi }+ \phi y_n$ in (17) and (20), respectively, both produce the exact posteriors when $h=0$. Both summary statistics are predictive sufficient in the weaker sense. In fact, the former is sufficient for *c* and $y_{n+p}$ for any $p\ge 1$ while the latter is that for $y_{n+1}$. Similarly as with (18), one can show that $\mathring{y}_{\phi }$ is not a good summary statistic for estimating *c*. The emergence of the statistic $y_n$ is not surprising given the Markov property of the model.

**Fig. 1:**
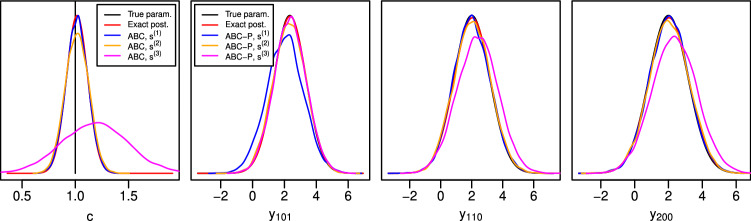
Illustration of the effect of summary statistics $s^{(1)}(y)=\bar{y}_{\phi }$, $s^{(2)}(y)=(\bar{y}_{\phi },y_n)$ and $s^{(3)}(y)=\mathring{y}_{\phi }=\bar{y}_{\phi }+\phi y_n$ on the ABC approximation accuracy in Example 3.1. *The first plot* on the left shows the ABC(-P/F) posterior for *c* and *the three other plots* the ABC-P posterior predictive distribution at some future time points. The black vertical line shows the true value of *c* used to generate the data and the red lines show the exact Gaussian posteriors obtained using (16) and (B.31) of Supplementary material with $h=0$

We also compute

21 $$\begin{aligned} \begin{aligned} \widehat{\pi }^{\,\smash {\text {(F)}}}_{h}(y_{n+1} \,|\,y; \bar{y}_{\phi })&= \widehat{\pi }^{\,\smash {\text {(F)}}}_{h}(y_{n+1} \,|\,y_n; \bar{y}_{\phi })\\&= \int _{-\infty }^{\infty } \pi (y_{n+1}\,|\,y_{n},c) \widehat{\pi }_h(c\,|\,\bar{y}_{\phi }) \mathop {}\!\textrm{d}c \\&= {{\,\mathrm{\mathcal {N}}\,}}(y_{n+1} \,|\,\mathring{y}_{\phi }, \sigma ^2 + \sigma ^2/n + h^2), \end{aligned} \end{aligned}$$

which shows that $\widehat{\pi }^{\,\smash {\text {(F)}}}_{0}(y_{n+1} \,|\,y; \bar{y}_{\phi })=\pi (y_{n+1} \,|\,y)$, as expected. We see that even a fairly large bandwidth *h* may not substantially deteriorate the quality of the ABC-F posterior predictive: Heuristically comparing the ABC posterior for *c* in (16) and the ABC-F posterior predictive for $y_{n+1}$ in (21) reveals that *h* needs to be small as compared to $\sigma ^2/n$ in the former case whereas as compared to $(1+1/n)\sigma ^2 \gg \sigma ^2/n$ in the latter case. We also see that ABC-F in (21) and ABC-P based on $\mathring{y}_{\phi }$ in (20) have the same target ABC posterior predictive. These methods are however based on different summary statistics and hence require different choices of *h* to yield similar computational efficiency in practice.

Finally, we analyze the summary statistics $s^{(1)}(y) ~{:}{=}~ \bar{y}_{\phi }$, $s^{(2)}(y) ~{:}{=}~ (\bar{y}_{\phi },y_n)$ and $s^{(3)}(y) ~{:}{=}~ \mathring{y}_{\phi }$ empirically in a more practical setting where the common uniform kernel with threshold *h* is now used. We consider $n=100$ observations and set $\phi =0.5$ and $\sigma ^2=1$. Another choice is considered in Supplementary material C.1. The true value of *c* is 1. The inference is carried out using ABC-MCMC with $10^6$ simulations (see Sect. 4 for details) and thresholds adjusted so that the acceptance probabilities are roughly $10\%$ for all three cases.

The results in Fig. 1 agree with the theoretical analysis above. In particular, we see that summary statistic $s^{(3)}$, which is sufficient only for predicting ${y}_{101}$, does not produce accurate posterior for *c*. While both $s^{(1)}$ and $s^{(2)}$ are sufficient for *c*, $s^{(1)}$ produces slightly more accurate approximation which is likely because matching this one-dimensional statistic is computationally more efficient than the two-dimensional $s^{(2)}$. As expected, $s^{(1)}$ produces less accurate posterior predictive at $t=101$ as the other summaries but unexpectedly works fairly well when $t=110$ and $t=200$. The ABC-F posterior predictive densities with $s^{(1)}$ and $s^{(2)}$ are not shown for clarity and because they both were essentially the same as the exact posterior. (Statistic $s^{(3)}$ was not considered as it would be a meaningless choice for ABC-F.)

#### A remark on computational efficiency

Computational efficiency of ABC is often a concern and this is especially the case with ABC-P. While matching of some “global” features of the data may be enough for accurate ABC parameter estimation, ABC-P might additionally require matching of some specific “local” features. Such “local” summary statistic for prediction, even if low-dimensional, can have high variability so that a large number of model simulations even with the “true” parameter may be needed to obtain a discrepancy evaluation that is small enough to ensure accurate posterior predictive distribution. For instance, consider the Markov model in Example 3.1 but with the summary statistic $\tilde{s}(y_{1:n})=y_n$ alone. In Supplementary material B.3, we show that for any $n\ge 1$, any fixed observations $y_{1:n}$ and threshold $\tilde{h}>0$, we have

22 $$\begin{aligned} \max _{c\in \mathbb {R}}\mathbb {P}(|z_n-y_n|\le \tilde{h}\,|\,c) \, {\left\{ \begin{array}{ll} \le \frac{\sqrt{2}\tilde{h}}{\sqrt{\pi \sigma ^2}}{\frac{\sqrt{\phi ^2-1}}{\sqrt{\phi ^{2n}-1}}}, &{} \text {if } |\phi |>1, \\ \le \frac{\sqrt{2}\tilde{h}}{\sqrt{\pi \sigma ^2}\sqrt{n}}, &{} \text {if } |\phi |=1, \\ \ge \frac{\sqrt{2}\tilde{h}}{\sqrt{\pi \sigma ^2}}\sqrt{1-\phi ^2}e^{-\tilde{h}^2/(2\sigma ^2)}, &{} \text {if } |\phi |<1, \end{array}\right. }\nonumber \\ \end{aligned}$$

where $\mathbb {P}(\cdot )$ is wrt. simulated data *z* given parameter *c*. It follows from (22) that the maximum probability of simulating data that matches the observation $y_n$ up to the absolute error $\tilde{h}$ goes to zero as $n\rightarrow \infty$ when $|\phi |\ge 1$. So, when the data size increases either substantially more simulations are needed or a larger error $\tilde{h}$ must be tolerated. The result is unsurprising because the model is a random walk when $|\phi | \ge 1$. On the other hand, the maximum acceptance probability is bounded from below with a positive constant in the stationary case $|\phi |<1$.

### Conditional predictive density with partial parameter dependence

We make some remarks on ABC prediction with ABC-P and ABC-F when the conditional predictive $\pi (\tilde{y}\,|\,y,\theta )$ depends only partially on $\theta$ and the goal is to estimate only $\tilde{y}$. We first suppose $\pi (\tilde{y}\,|\,y,\theta ) = \pi (\tilde{y}\,|\,y)$. The first condition of predictive sufficiency (12) then becomes irrelevant and the second condition (13) is the same as the weaker form of predictive sufficiency (11). Hence, in a (hypothetical) situation where this special condition holds but one can nevertheless only simulate jointly from $\pi (\tilde{y},y\,|\,\theta )$, ABC-P is otherwise implemented as before but the summary statistic used does not need to be informative about $\theta$. Additionally, ABC-F reverts to direct simulation from $\pi (\tilde{y}\,|\,y)$ in this case. A simple analytical example would be $y_t\,|\,\theta \sim _{\,\text {i.i.d.}}{{\,\mathrm{\mathcal {N}}\,}}(\theta ,1^2)$ for $t=1,\ldots ,n$ and $\tilde{y}=y_{n+1}$ such that $y_{n+1}\,|\,y, \theta \sim {{\,\mathrm{\mathcal {N}}\,}}(y_{n+1}\,|\,y_n,1^2)$. Then it clearly holds that $\pi (\tilde{y}\,|\,y,\theta ) = \pi (\tilde{y}\,|\,y) = \pi (\tilde{y}\,|\,y_n)$. A statistic $s$ is called *purely predictive sufficient* in Bjørnstad (1996, Section 3.1) if $\pi (\tilde{y}\,|\,y,\theta ) = \pi (\tilde{y}\,|\,s_y)$ which implies $\pi (\tilde{y}\,|\,y,\theta ) = \pi (\tilde{y}\,|\,y)$. In the example, $y_n$ is purely predictive sufficient.

We next consider a situation more relevant for practical applications. We write $\theta =(\psi ,\eta )$ and suppose $\pi (\tilde{y}\,|\,y,\theta ) = \pi (\tilde{y}\,|\,y,\psi )$, that is, the conditional predictive density depends only on some of the parameters so that

23 $$\begin{aligned} \pi (\tilde{y}\,|\,y)= & {} \iint \pi (\tilde{y}\,|\,y,\psi ) \pi (\psi ,\eta \,|\,y)\mathop {}\!\textrm{d}\psi \mathop {}\!\textrm{d}\eta \nonumber \\= & {} \int \pi (\tilde{y}\,|\,y,\psi ) \pi (\psi \,|\,y)\mathop {}\!\textrm{d}\psi . \end{aligned}$$

For instance, $\eta$ could be a parameter of a noise model which is not assumed for future data $\tilde{y}$. A simple analytical example would be $y_t\,|\,y_{t-1}, \theta \sim {{\,\mathrm{\mathcal {N}}\,}}(\psi +y_{t-1},\eta ^2)$ for $t=1,\ldots ,n$, $(\psi ,\eta )\in \mathbb {R}\times \mathbb {R}_{+}$ and $\tilde{y}=y_{n+1}$ such that $y_{n+1}\,|\,y_n,\theta \sim {{\,\mathrm{\mathcal {N}}\,}}(y_{n+1}\,|\,\psi +y_n,1^2)$. When (23) holds, it is in principle enough that the summary statistic *s* is informative for $\psi$ in ABC-F. Similarly, in the case of ABC-P, the predictive sufficiency conditions (12) and (13) need to concern only $\psi$ instead of $\theta$. In other words, in both cases, $\pi (\eta \,|\,\psi , y)$ in principle does not need to be accurately approximated, only $\pi (\psi \,|\,y)$ does. Design of informative low-dimensional summary statistics for ABC approximation of marginal posteriors, such as $\pi (\psi \,|\,y)$ in our above case, has recently been studied by Drovandi et al. (2022).

### Tractable simulation from conditional predictive given latent variables

We have now considered ABC prediction when simulation either from both $\pi (\tilde{y}\,|\,y,\theta )$ and $\pi (y\,|\,\theta )$, or jointly from $\pi (\tilde{y},y\,|\,\theta )$, is feasible. In this section we briefly study an alternative that takes advantage of the latent variable representation

24 $$\begin{aligned} \pi (\tilde{y}\,|\,y, \theta ){} & {} = \int \pi (\tilde{y},v\,|\,y, \theta ) \mathop {}\!\textrm{d}v \\ \nonumber{} & {} = \int \pi (\tilde{y}\,|\,y, v, \theta ) \pi (v \,|\,y,\theta ) \mathop {}\!\textrm{d}v, \end{aligned}$$

where *v* denotes finite-dimensional, unobserved latent variables involved in the model.

Combining (24) and (1) produces

25 $$\begin{aligned} \pi (\tilde{y}\,|\,y) = \iint \pi (\tilde{y}\,|\,y, v, \theta ) \pi (v,\theta \,|\,y) \mathop {}\!\textrm{d}v \mathop {}\!\textrm{d}\theta , \end{aligned}$$

which motivates the following approximation of the posterior predictive density:

26 $$\begin{aligned} \pi (\tilde{y}\,|\,y)&\approx \widehat{\pi }^{\,\smash {\text {(L)}}}_h(\tilde{y}\,|\,y;s_y) \nonumber \\&~{:}{=}~ \iint \pi (\tilde{y}\,|\,y, v, \theta ) \widehat{\pi }_h(v,\theta \,|\,s_y) \mathop {}\!\textrm{d}v \mathop {}\!\textrm{d}\theta , \end{aligned}$$

27 $$\begin{aligned} \widehat{\pi }_h(v,\theta \,|\,s_y)&~{:}{=}~ \frac{\int K_h({\Delta (s_y,s_z)}) \pi (s_z,v\,|\,\theta ) \pi (\theta ) \mathop {}\!\textrm{d}s_z}{\iiint K_h({\Delta (s_y,s_z)}) \pi (s_z,v\,|\,\theta ) \pi (\theta ) \mathop {}\!\textrm{d}s_z \mathop {}\!\textrm{d}\theta \mathop {}\!\textrm{d}v}. \end{aligned}$$

This approach is called ABC-L where “L” refers to the latent variables. To implement ABC-L, the intractable model under consideration must satisfy (24) and sampling from both $\pi (\tilde{y}\,|\,y, v, \theta )$ and $\pi (y, v\,|\,\theta )$ must be feasible. The latter assumption is evidently weaker than that of ABC-F: If one can directly sample from $\pi (\tilde{y}\,|\,y, \theta )$, then one can also sample from $\pi (\tilde{y}\,|\,y, v, \theta )$ by formally setting $v=\varnothing$. Note that $\pi (v\,|\,y,\theta )$ is not assumed tractable—if one can additionally sample from $\pi (v\,|\,y,\theta )$ then one can also sample from $\pi (\tilde{y}\,|\,y, \theta )$ using (24) so that ABC-F applies. This is the case in Frazier et al. (2019, Section 4) where ABC-F was used for SMMs. ABC-L is in fact similar to ABC-F if $\theta$ is allowed to contain latent variables, that is, if $\theta$ is replaced with $(v,\theta )$. However, the asymptotic results of Frazier et al. (2019) do not apply for ABC-L as such because the latent variables *v* cannot typically be consistently estimated.

One can establish that $\widehat{\pi }_{h_t}(v,\theta \,|\,s_y)\rightarrow \pi (v,\theta \,|\,s_y)$ as $t\rightarrow \infty$ but we omit the details as they are analogous to Sect. 3.2.1 and as a similar result is obtained as a special case of Jasra (2015, Proposition 2.1). Clearly, the summary statistic $s$ should ideally be jointly sufficient for *v* and $\theta$ so that

28 $$\begin{aligned} \pi (v,\theta \,|\,y) = \pi (v,\theta \,|\,s_y). \end{aligned}$$

This condition resembles Bayes sufficiency. Similarly as discussed in Sect. 3.3, $\pi (\tilde{y}\,|\,y, v, \theta )$ may depend only on some components of $(v,\theta )$ so that the sufficiency condition (28) in principle needs to hold only for them. Similarly to the case of ABC-F, the ABC-L posterior predictive (26) agrees with the exact posterior predictive $\pi (\tilde{y}\,|\,y)$ if $\widehat{\pi }_h(v,\theta \,|\,s_y) = \pi (v,\theta \,|\,y)$.

A key difference between ABC-L and ABC-P is that latter method depends on the full data *y* only via the summary statistic $s_y$ while the former approach, similarly to ABC-F, depends directly on *y* via the conditional predictive $\pi (\tilde{y}\,|\,y, v, \theta )$ in (26). A potential downside of ABC-L is that, even when one could use the fact that the conditional predictive depends only on some components of $(v,\theta )$, ABC inference in high-dimensional space might be needed which is notoriously a hard problem.

We end this section with a SSM example that demonstrates the selection of summary statistics for ABC-P and ABC-L. Nonetheless, in practice ABC-L should be used for truly intractable models that are different from the basic SSM defined via (3). Namely, in this case $\pi (\tilde{y}\,|\,y, v, \theta ) = \pi (\tilde{y}\,|\,v_n, \theta )$ does not depend on *y* and it can be easily checked that ABC-P and ABC-L then in fact have the same target ABC posterior predictive.

#### Example 3.2

(State space model) Consider the following model

29 $$\begin{aligned} v_t\,|\,v_{t-1},\theta \sim {{\,\mathrm{\mathcal {N}}\,}}(c+\phi v_{t-1},\sigma ^2), \quad y_t\,|\,v_t,\theta \sim {{\,\mathrm{\mathcal {N}}\,}}(v_t,\omega ^2), \nonumber \\ \end{aligned}$$

where $t=1,2,\ldots ,n$ with $n>1$ and $v_0=0$. The set-up is similar to Example 3.1 except that the (latent) states are denoted by *v* and are not observed. Also, $\omega >0$ is an additional fixed parameter. We consider only the case $h=0$ and investigate the effect of the summary statistics for estimating *c* or $\tilde{y}=y_{t+1}$ given observations $y=y_{1:n}$. Justifications for the various results below are given in Supplementary material B.4.

Consider the following scalar-valued summary statistics $s^{(1)}(y_{1:n}) ~{:}{=}~ \mu ^{\top }W^{-1}y_{1:n}$ and $s^{(2)}(y_{1:n}) ~{:}{=}~ \Sigma _{n:} W^{-1}y_{1:n}$. Here $\mu \in \mathbb {R}^n$ is such that $\mu _t = (1-\phi ^t)/(1-\phi )$, $\Sigma \in \mathbb {R}^{n\times n}$ is the covariance matrix of *v* that depends on $\phi$ and $\sigma ^2$ (but not on *c*) and whose formula is given in (B.24) of Supplementary material B.4, $\Sigma _{n:}$ denotes the *n*th row of $\Sigma$ and $W=\Sigma + \omega ^2\text {I}$, where $\text {I}\in \mathbb {R}^{n\times n}$ is the identity matrix. First of all, $s^{(1)}(y_{1:n})$ is minimal parametric sufficient for *c* and a linear combination of $s^{(1)}(y_{1:n})$ and $s^{(2)}(y_{1:n})$, shown in (B.53) of Supplementary material B.4, is minimal predictive sufficient for $y_{n+1}$. Hence, these would be ideal summary statistics for computing the ABC posterior of *c* and the ABC-P posterior predictive of $y_{n+1}$, respectively.

Access to $\pi (y_{n+1}\,|\,y_{1:n},v_{1:n},c)$, which here simplifies to $\pi (y_{n+1}\,|\,v_n,c)={{\,\mathrm{\mathcal {N}}\,}}(c+\phi v_n,\omega ^2+\sigma ^2)$, and consequently the ABC posterior of $(v_n,c)$ would be both required for ABC-L. Now, $(s^{(1)}(y_{1:n}),s^{(2)}(y_{1:n}))$ qualifies for a sufficient statistic for $(v_n,c)$ in the sense of (28) and is hence sufficient also for $y_{n+1}$ in the ABC-L approach. This statistic is two-dimensional which is not surprising as it is sufficient for $(v_n,c)\in \mathbb {R}^2$ in a Gaussian linear system. As $\pi (y_{n+1}\,|\,y_{1:n},v_{1:n},c)$ does not depend on $y_{1:n}$ and depends on $(v_{1:n},c)$ only via $c+\phi v_n$, it can be argued that the same scalar-valued summary statistic that is predictive sufficient for $y_{n+1}$, is also sufficient for $y_{n+1}$ in the ABC-L approach. This shows that $(s^{(1)}(y_{1:n}),s^{(2)}(y_{1:n}))$ is not minimal sufficient in this case.

## Using ABC samplers for predictive inference

We next consider numerical methods for ABC-L and ABC-P. Our aim is not to develop or advocate some specific algorithm but instead concisely outline how commonly used ABC algorithms can be modified to produce samples from the ABC-L and ABC-P target densities. We also discuss some practical aspects of predictive ABC inference.

### Sampling from ABC posterior predictive

We assume the observed summary statistic $s_y$, discrepancy $\Delta$, kernel $K_h$ and threshold $h>0$ are all fixed and consider first the task of sampling from $\widehat{\pi }_h(\theta \,|\,s_y)$ in (5). The ABC target density is augmented to $\widehat{\pi }_h(s_z,\theta \,|\,s_y) \propto K_h({\Delta (s_y,s_z)}) \pi (s_z\,|\,\theta ) \pi (\theta )$ and the proposal density selected as $\pi (s_z\,|\,\theta )q(\theta )$. Self-normalized importance sampling (IS) can be then used as the resulting (unnormalized) importance weights are free of intractable terms:

30 $$\begin{aligned} \tilde{\omega }(\theta ) = \frac{K_h({\Delta (s_y,s_z)}) {\pi (s_z\,|\,\theta )} \pi (\theta )}{{\pi (s_z\,|\,\theta )}q(\theta )} = \frac{K_h({\Delta (s_y,s_z)}) \pi (\theta )}{q(\theta )}. \end{aligned}$$

Weighted samples $(\tilde{\omega }^{(i)},\theta ^{(i)})$ from $\widehat{\pi }_h(\theta \,|\,s_y)$ are hence obtained by first sampling $\theta ^{(i)}$ from $q(\theta )$, then $z^{(i)}$ from $\pi (z\,|\,\theta ^{(i)})$, and finally computing $\tilde{\omega }^{(i)} = K_h({\Delta (s_y,s_z^{\smash {(i)}})}) \pi (\theta ^{(i)}) / q(\theta ^{(i)})$ with $s_z^{\smash {(i)}}=s(z^{(i)})$ for $i=1,\ldots ,m$. Normalized weights ${\omega }^{(i)}$ can be obtained by computing $\omega ^{(i)}=\tilde{\omega }^{(i)}/\sum _{j=1}^m\tilde{\omega }^{(j)}$.

We similarly define the augmented target density $\widehat{\pi }^{\,\smash {\text {(L)}}}_h(s_z,v,\theta \,|\,s_y) \propto K_h({\Delta (s_y,s_z)}) \pi (s_z,v\,|\,\theta ) \pi (\theta )$ for the ABC-L approach. If the proposal is analogously set to $\pi (s_z,v\,|\,\theta )q(\theta )$, the intractable terms $\pi (s_z,v\,|\,\theta )$ cancel out similarly as $\pi (s_z\,|\,\theta )$ in (30) and the same (unnormalized) importance weight $\tilde{\omega }(\theta )$ results. Hence, weighted samples $(\tilde{\omega }^{(i)},\tilde{y}^{(i)})$ from $\widehat{\pi }^{\,\smash {\text {(L)}}}_h(\tilde{y}\,|\,y;s_y)$ in (26) are obtained by repeating the following steps

1. Simulate $$\theta ^{(i)}$$ from $$q(\theta )$$,
2. Simulate $(z^{(i)},v^{(i)})$ from $\pi (z,v\,|\,\theta ^{(i)})$ and compute $s_z^{\smash {(i)}}=s(z^{(i)})$,
3. Compute (unnormalized) IS weight $\tilde{\omega }^{(i)} ={K_h({\Delta (s_y,s_z^{\smash {(i)}}})}) \pi (\theta ^{(i))} /q(\theta ^{(i)})$,
4. Simulate $$\tilde{y}^{(i)}$$ from $$\pi (\tilde{y}\,|\,y, v^{(i)}, \theta ^{(i)})$$,

for $i=1,\ldots ,m$. Alternatively, a two stage approach can be used where steps L1-L3 are first repeated for all *i* and L4 is finally run for those samples with non-zero weights. ABC rejection sampler for ABC-L results as a special case with $K_h(r) \propto \mathbb {1}_{r\le h}$ and $q(\theta )=\pi (\theta )$. Up to some caveats discussed later, PMC-ABC (Beaumont et al. 2009) and other related sequential IS methods such as Sisson et al. (2007), Toni et al. (2009), Del Moral et al. (2012) can be similarly modified to sample from the (augmented) ABC-L target. This is also the case with ABC-MCMC (Marjoram et al. 2003). All in all, the main modifications are that also $v^{(i)}$ need to be stored (unless e.g. its weight is zero) and the extra prediction step L4.

Sampling from the ABC-P target $\widehat{\pi }^{\,\smash {\text {(P)}}}_{h}(\tilde{y} \,|\,s_y)$ in (8) proceeds in a similar fashion as above except that future data $\tilde{y}$ is already drawn alongside *z* in L2 so that there is no specific prediction step L4. For a change, we consider ABC-MCMC sampler. The augmented ABC-P target density is $\widehat{\pi }^{\,\smash {\text {(P)}}}_h(\tilde{y},s_z,\theta \,|\,s_y) \propto K_h({\Delta (s_y,s_z)}) \pi (\tilde{y},s_z\,|\,\theta ) \pi (\theta )$ and the proposal density is $g((\tilde{y},s_z,\theta ),(\tilde{y}^*,s_z^*,\theta ^*)) ~{:}{=}~ \pi (\tilde{y}^*,s_z^*\,|\,\theta ^*)q(\theta ^*\,|\,\theta )$, where $(\tilde{y},s_z,\theta )$ denotes the current state of the chain and $(\tilde{y}^*,s_z^*,\theta ^*)$ the proposed state. The Metropolis-Hastings acceptance probability for a transition from $(\tilde{y},s_z,\theta )$ to $(\tilde{y}^*,s_z^*,\theta ^*)$ is then

31 $$\begin{aligned} \begin{aligned}&\alpha ((\tilde{y},s_z,\theta ),(\tilde{y}^*,s_z^*,\theta ^*))\\&\quad = \min \left\{ 1, \frac{K_h({\Delta (s_y,s_z^*)}) \pi (\tilde{y}^*,s_z^*\,|\,\theta ^*) \pi (\theta ^*)}{K_h({\Delta (s_y,s_z)}) \pi (\tilde{y},s_z\,|\,\theta ) \pi (\theta )} \, \times \,\frac{g((\tilde{y}^*,s_z^*,\theta ^*),(\tilde{y},s_z,\theta ))}{g((\tilde{y},s_z,\theta ),(\tilde{y}^*,s_z^*,\theta ^*))} \right\} \\&\quad = \min \left\{ 1, \frac{K_h({\Delta (s_y,s_z^*)}) \pi (\theta ^*) q(\theta \,|\,\theta ^*)}{K_h({\Delta (s_y,s_z)}) \pi (\theta ) q(\theta ^*\,|\,\theta )} \right\} . \end{aligned} \end{aligned}$$

Starting from an initial point $(s_z^{\smash {(0)}},\theta ^{(0)})$ (which should be such that $K_h({\Delta (s_y,s_z^{\smash {(0)}})}) \pi (\theta ^{(0)})>0$), correlated samples from $\widehat{\pi }^{\,\smash {\text {(P)}}}_{h}(\tilde{y},\theta \,|\,s_y)$ are generated by iterating the following steps for $i=1,\ldots ,m$:

1. Simulate $$\theta ^*$$ from $$q(\theta \,|\,\theta ^{(i-1)})$$,
2. Simulate $(\tilde{y}^*,z^*)$ from $\pi (\tilde{y},z\,|\,\theta ^*)$ and compute $s_z^*=s(z^*)$,
3. Set $(\tilde{y}^{(i)},z^{(i)},\theta ^{(i)}) = (\tilde{y}^*,z^*,\theta ^*)$ with probability $\alpha ((\tilde{y}^{(i-1)},s_z^{\smash {(i-1)}},\theta ^{(i-1)}),(\tilde{y}^*,s_z^*,\theta ^*))$ using (31) and otherwise set $(\tilde{y}^{(i)},z^{(i)},\theta ^{(i)}) = (\tilde{y}^{(i-1)},z^{(i-1)},\theta ^{(i-1)})$.

Analogously to standard ABC-MCMC with the augmented target density $\widehat{\pi }_h(s_z,\theta \,|\,s_y)$, the detailed balance condition can be shown to hold so that $\widehat{\pi }^{\,\smash {\text {(P)}}}_h(\tilde{y},s_z,\theta \,|\,s_y)$ is indeed the stationary distribution of the chain generated by the above algorithm.

The acceptance probability (31) and the corresponding IS weight do not depend on $\tilde{y}$. Consequently, the computation time of ABC-P can be reduced using the latent variable representation

32 $$\begin{aligned} \pi (\tilde{y},z\,|\,\theta ) = \int \pi (\tilde{y}\,|\,z, v, \theta ) \pi (v,z \,|\,\theta ) \mathop {}\!\textrm{d}v, \end{aligned}$$

which is the same condition as (24) assumed for ABC-L. Specifically, one then first simulates $(z^{(i)},v^{(i)},\theta ^{(i)})$ for each *i* and afterwards simulates only those of the corresponding predictions $\tilde{y}^{(i)}$ using $\pi (\tilde{y}\,|\,z^{(i)}, v^{(i)}, \theta ^{(i)})$ that are required to form the final estimate of $\pi (\tilde{y}\,|\,y)$. For example, those $\tilde{y}^{(i)}$ that correspond to “burn-in” in the ABC-MCMC sampler or that either have zero IS weight or do not belong to the last population in the PMC-ABC algorithm, are not required. In some cases jointly sampling $\tilde{y}$ and *z* is not much slower than sampling only *z*. The above approach can still be useful because the samples can be reused for other related prediction tasks for which the selected summary statistic is deemed informative.

Adapting some special ABC samplers for ABC-L and ABC-P may require additional modifications. Examples include the adaptive method for adjusting the discrepancy by Prangle (2017), which is further discussed in Sect. 4.2, and the adaptive threshold selection method by Simola et al. (2021), which could possibly be modified to account for the posterior predictive at least when $\tilde{y}$ is low-dimensional.

### On forming the discrepancy for predictive ABC inference

A common choice for the discrepancy is the weighted Euclidean distance between the summary statistics. Its weights are often based on the variability of the summary statistic which is estimated using pilot simulations (or adjusted adaptively as in Prangle (2017)). However, such an approach may be unsuitable for ABC-P. For example, consider the Markov model of Example 3.1 where the summary statistic $\bar{y}_{\phi }$ is informative for the model parameter *c* and the statistic $y_n$, the last observation, is informative for prediction. We formed a discrepancy $\Delta (s_y,s_z)=(\bar{y}_{\phi }-\bar{z}_{\phi })^2/\mathbb {V}(\bar{z}_{\phi }) + (y_n-z_n)^2/\mathbb {V}(z_n)$ where the variances were estimated via pilot simulations. We then observed that this common approach lead to too low weight for the statistic $y_n$ due to its fairly high variability (see Sect. 3.2.3) and consequently noticeable ABC error in the prediction. This also happened when e.g. robust measures of variability were used and in the more realistic case of the continuous-time Markov model of Sect. 5.2. This difficulty can be alleviated by manual readjustment but this is a cumbersome and opaque procedure in practice.

The use of multiple discrepancies (see Sect. 2.2) can be helpful for appropriately weighting the summary statistics in some ABC prediction situations. Suppose that a meaningful partition of the summary statistic exists so that $s(y)=(\bar{s}(y),\tilde{s}(y))$ where $\bar{s}(y)$ denotes those statistics that are deemed informative for the parameter $\theta$, and $\tilde{s}(y)$ those that are deemed informative for prediction (and ideally also interpretable). In our Markov example these would be $\bar{s}(y)=\bar{y}_{\phi }$ and $\tilde{s}(y)=y_n$. Then one could use the kernel

33 $$\begin{aligned}{} & {} \mathbb {1}_{\left\| \bar{s}_y-\bar{s}_z\right\| \le \bar{h}_t} \mathbb {1}_{\left\| \tilde{s}_y-\tilde{s}_z\right\| ' \le \tilde{h}_t} \nonumber \\{} & {} \quad = \mathbb {1}_{s_z\in \left\{ u=(\bar{u},\tilde{u})\in \mathbb {R}^d: \left\| \bar{s}_y-\bar{u}\right\| \le \bar{h}_t, \left\| \tilde{s}_y-\tilde{u}\right\| ' \le \tilde{h}_t\right\} }, \end{aligned}$$

where $\bar{h}_t$ and $\tilde{h}_t$ denote separate thresholds and $\Vert \cdot \Vert$, $\Vert \cdot \Vert '$ the corresponding norms. If one chooses $\bar{h}_t/a=\tilde{h}_t/b=h_t$ where $a,b>0$ are fixed constants and $h_t$ a sequence of thresholds such that $h_t\rightarrow 0$ as $t\rightarrow \infty$, then the acceptance region of (33) can be written as $\mathcal {A}_t=\{u=(\bar{u},\tilde{u})\in \mathbb {R}^d:\max \{a\Vert \bar{s}_y-\bar{u}\Vert ,b\Vert \tilde{s}_y-\tilde{u}\Vert '\}\le h_t\}$. It is easy to check that $\mathcal {A}_t$ satisfies the assumptions (C2), (C4) and (C5) so that Proposition 3.1 applies. An important technical condition is that $\bar{h}_t$ and $\tilde{h}_t$ decay at the same rate, otherwise the assumption (C5) would not hold. In our experiments in Sect. 5 where the above approach is used, $||\cdot ||'$ and $\tilde{h}_t$ are selected based on expert knowledge and the scale of the observed data, respectively. The weights for $\Vert \cdot \Vert$ can be determined using pilot runs or adaptively. The best practices likely depend on the problem at hand and a detailed investigation falls outside of the scope of this paper.

## Illustrative examples

We next demonstrate the predictive ABC methods of Sect. 3 using two intractable dynamic models, M/G/1 queue (Sect. 5.1) and stochastic Lotka–Volterra (Sect. 5.2). The goal of these numerical experiments is two-fold: First, to show that ABC-P and ABC-L can produce reasonable approximations of the posterior predictive distribution in practice, although overestimated uncertainty often results as is common also in the standard ABC inference. Second, the results indicate that the goal of prediction should be accounted for when designing the summary statistics, although informative low-dimensional summary statistics (both for parameter estimation and prediction) are challenging to design for realistic applications. While some insight on selecting informative summaries and suitable ABC sampler configurations for the goal of prediction is provided, we do not aim for a comprehensive analysis. This is because these choices depend on the problem at hand and are active topics of ongoing research also in the context of standard ABC inference.

ABC-F and ABC-L approaches do not apply in all of our test cases unlike ABC-P which, in principle, can be implemented whenever jointly sampling from $\pi (\tilde{y},y\,|\,\theta )$ is feasible. Importantly, all three approaches produce the standard ABC posterior for $\theta$ as their marginal whenever they can be implemented. Hence, whenever we additionally computed this quantity, we only show it for ABC-P and the reader needs to notice that ABC-F and ABC-L based on the same summary statistic, discrepancy and threshold as ABC-P would also produce the same result. R-code (with fast C-implementation for model simulation) used for the experiments is available at https://github.com/mjarvenpaa/ABC-pred-inf.

### M/G/1 queue model

We first consider the M/G/1 queue model where customers arrive at a single server with independent interarrival times $w_i\sim {{\,\textrm{Exp}\,}}(\theta _3)$, that is, the arrival times follow Poisson process with rate parameter $\theta _3$. The queue is empty before the first arrival. The service times $u_i$ are assumed to be independently distributed so that $u_i\sim {{\,\mathrm{\mathcal {U}}\,}}([\theta _1,\theta _2])$. Only the interdeparture times $y=(y_1,\ldots ,y_n)$ are observed. This model has been used before e.g. by Fearnhead and Prangle (2012); Jiang et al. (2018); Bernton et al. (2019) to demonstrate ABC algorithms for estimating the model parameters $\theta =(\theta _1,\theta _2,\theta _3)$. We also assume that the parameters $\theta$ are unknown but the main task is to estimate the waiting times of future customers.

The interdeparture times $y_i$ satisfy

34 $$\begin{aligned} y_i{} & {} = u_i + \max \bigg \{ 0, \sum _{j=1}^i w_j - \sum _{j=1}^{i-1} y_j \bigg \} \\ \nonumber{} & {} = u_i + \max \left\{ 0, v_i - x_{i-1} \right\} , \end{aligned}$$

where $v_i~{:}{=}~\sum _{j=1}^i w_j$ are the arrival times and $x_i~{:}{=}~\sum _{j=1}^i y_j$ the corresponding departure times. We use the convention $v_0=x_0=0$. While the likelihood function $\pi (y\,|\,\theta )$ is intractable as discussed by Heggland and Frigessi (2004), using (34) and as $v_i=v_{i-1}+w_i, w_i \sim {{\,\textrm{Exp}\,}}(\theta _3)$, one can easily simulate from

35 $$\begin{aligned}{} & {} \pi (y_i,v_i\,|\,y_{1:i-1},v_{1:i-1},\theta ) = \pi (y_i,v_i\,|\,x_{i-1},v_{i-1},\theta ) \nonumber \\{} & {} \quad = \pi (y_i\,|\,x_{i-1},v_i,\theta _{1:2})\pi (v_i\,|\,v_{i-1},\theta _3) \end{aligned}$$

and hence from $\pi (y,v\,|\,\theta )$, where $v=(v_1,\ldots ,v_n)$. Obviously, this way one can sample also from $\pi (\tilde{y},y\,|\,\theta )$ where $\tilde{y}=(y_{n+1},\ldots ,y_{n+\tilde{n}})$ denote the interarrival times of the next $\tilde{n}$ future customers.

We use $\omega _i ~{:}{=}~ x_i-v_i, i=1,\ldots ,n$, to denote the waiting times of customers and similarly $\tilde{\omega }_i, i = n+1,\ldots ,n+\tilde{n}$, denote those of the future customers. This definition includes both the time the customer waits to be served (which can be 0) and the service time. Clearly, sampling from $\pi (\tilde{\omega },y\,|\,\theta )$ is feasible so ABC-P can be easily implemented. Similarly, simulating from $\pi (\tilde{\omega }\,|\,y,v,\theta ) = \pi (\tilde{\omega }\,|\,x_n,v_n,\theta )$ is feasible which facilitates ABC-L. Simulating from $\pi (\tilde{y}\,|\,y, \theta )$ or $\pi (\tilde{\omega }\,|\,y, \theta )$, and consequently implementing ABC-F, is not straightforward due to the latent variables *v* involved and is hence not considered.^6^

#### Common experimental details

We use the uniform prior $(\theta _1,\theta _2-\theta _1,\theta _3) \sim {{\,\mathrm{\mathcal {U}}\,}}([0,10]^2\times [0,1/3])$. In addition, we account for the constraint $\theta _1 \le \min _{i\in \{1,\ldots ,n\}}y_i$, which is is implicitly coded into the intractable likelihood function but not in the ABC procedure, as discussed by Bernton et al. (2019). Although M/G/1 model has commonly been used to study ABC algorithms, particle MCMC methods (Andrieu et al. 2010) and the special MCMC algorithm by Shestopaloff and Neil (2014) in principle also apply. We adopt the latter MCMC method and we further extend it in a straightforward way to obtain samples from $\pi (\tilde{\omega }\,|\,y)$. This allows us to compare the accuracy of the ABC methods to the ground-truth. We also consider the predictive distribution $\pi (\tilde{\omega }\,|\,y, v, \theta _{\text {true}})$ as this unveils the best possible prediction accuracy. Of course, in reality this baseline would be unavailable as $\theta _{\text {true}}$ is unknown and *v* is unobserved.

**Fig. 2:**
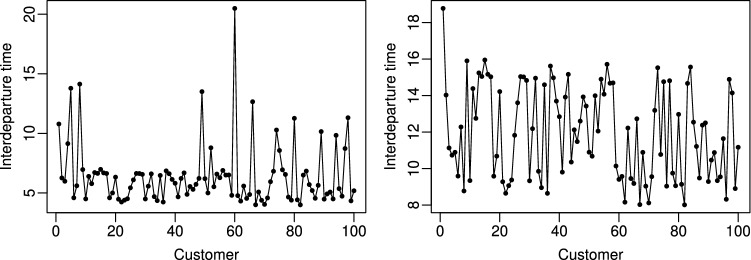
Observed interdeparture times $y=(y_1,\ldots ,y_n)$ with $n=100$ used in the M/G/1 experiments. *Left plot:* The case of Sect. 5.1.2 where the queue length is varying. Occasional large interdeparture times suggest that the queue could be then empty. *Right plot:* The case of Sect. 5.1.3 where the queue length tends to be growing

We form a common 5-dimensional baseline summary statistic $s^{(0)}$ by using the $\alpha =0.25$, 0.5 and 0.75th empirical quantiles $\hat{q}_{\alpha }(y)$, and the range of $y$. For comparison, we adopt the strategy outlined in Sect. 4.2 and form another summary statistic $s^{(1)}(y) = (\bar{s}^{(1)}(y),\tilde{s}^{(1)}(y))$, where $\bar{s}^{(1)}(y) = {s}^{(0)}(y)$ and where the additional summaries $\tilde{s}^{(1)}(y)$ are given by $\max \{i\ge 1: y_i\ge \hat{q}_{\tilde{\alpha }}(y_{\text {obs}})\}$ with convention $\max \varnothing =1$. Note that $y$ denotes any data realization while $\hat{q}_{\tilde{\alpha }}(y_{\text {obs}})$ is computed using the observed data at hand denoted here as $y_{\text {obs}}$ for clarity. This tentative summary statistic captures the idea that individual large interdeparture times result when the queue is empty and the locations of such recent values provide information about the state of the queue for prediction. We use this strategy and $s^{(1)}$ also for ABC-L. The statistic $\tilde{s}^{(1)}(y) = y_n$ is not chosen as it would be rather uninformative—short interdeparture times occur frequently irrespective of the state of the queue as seen in Fig. 2.

We use $\tilde{\alpha }=$ 0.7, 0.8 and 0.9 so that $\tilde{s}^{(1)}(y)\in \mathbb {R}^3$. We choose $||\cdot ||$ for both $s^{(0)}$ and $\bar{s}^{(1)}$ so that $||r|| = r^{\top }\hat{C}^{-1}r$ where $\hat{C}$ is the empirical covariance matrix of the summaries estimated, for simplicity and to facilitate meaningful comparison, by running the model $10^3$ times at $\theta _{\text {true}}$. This value would be unavailable in practice but other point estimates or the approach by Prangle (2017) could be then used instead. We choose $||\cdot ||'$ as the $L^{\infty }$-norm so that $||r||' = \max _{i\in \{1,2,3\}}|r_i|$. We use ABC-MCMC with $2\cdot 10^6$ iterations and burn-in $10^4$ in both cases. In the case of $s^{(0)}$, the threshold *h* is determined so that the acceptance probability (after burn-in) is $2\%$. In the case of $s^{(1)}$ we set $\tilde{h}=5$ (Sect. 5.1.2) or $\tilde{h}=7$ (Sect. 5.1.3). The threshold $\bar{h}$ is then determined to give the same acceptance rate as with $s^{(0)}$ to make the comparison meaningful although it is not possible to completely separate the effect of the summary statistic and choices related to computational efficiency.

#### Varying queue

We first consider a situation where $\theta _{\text {true}}=(4, 7, 0.15)$. In this case the queue length tends to vary being occasionally empty and occasionally containing several customers. A simulated data set with $n=100$ used in the experiments below is shown in Fig. 2.

**Fig. 3:**
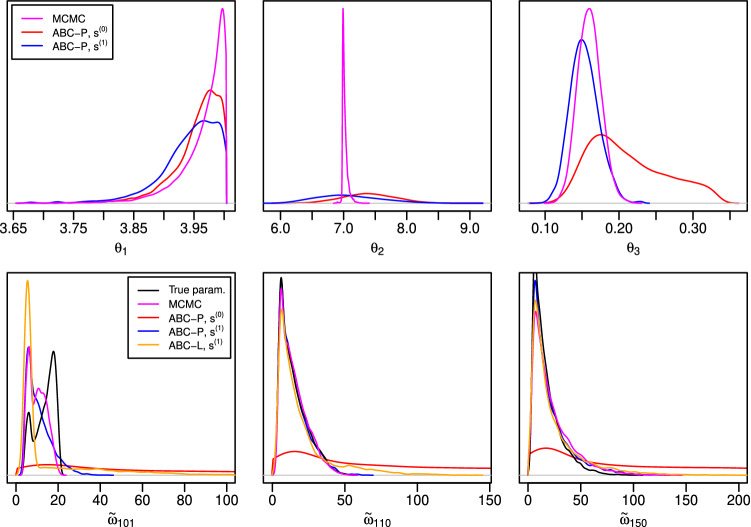
Results for the M/G/1 experiments of Sect. 5.1.2. *Top row:* Marginal posterior distributions computed using MCMC and ABC for parameters $\theta$. *Bottom row:* Predictive distribution based on the true parameter $\theta _{\text {true}}$ (“True param.”) and posterior predictive distributions computed using MCMC and ABC approaches for the waiting times of some future customers. The right tail of the ABC-P posterior predictive based on $s^{(0)}$ is truncated to ease visualization

The top row of Fig. 3 shows the ABC posteriors of the parameter $\theta$. The marginal ABC posteriors for $\theta _1$ are both fairly accurate, though the baseline summary statistic $s^{(0)}$ works slightly better. The likely reason is that the threshold $\bar{h}$ was set slightly larger than *h* because the additional summaries $\tilde{s}^{(1)}$ need also to be matched in this case and because the ABC-MCMC acceptance probability was required to be the same in both cases. On the other hand, $s^{(1)}$ works better for $\theta _3$ which suggests that the additional summaries $\tilde{s}^{(1)}$ designed for prediction are informative also for this parameter. As common also in other ABC analyses in literature, poor marginal posterior approximations for $\theta _2$ are observed.

The bottom row of Fig. 3 shows the corresponding results for the prediction task. Interestingly, the ideal predictive density and the exact posterior predictive for the first future waiting time $\omega _{101}$ are both bimodal. (We run several MCMC chains to ensure our observations are not caused by possible numerical issues.) An important result is that the ABC-P posterior predictive based on the baseline summary statistic $s^{(0)}$ has far too long right tail. This occurs because $s^{(0)}$ is invariant to the order of the observations and hence the trend of the observed interdeparture times just before the prediction gets lost. This would also be the outcome with other methods in literature such as the distance functions studied by Jiang et al. (2018), Bernton et al. (2019). ABC-L posterior predictive also has a pronounced right tail but the additional summaries $\tilde{s}^{(1)}$ appear informative for prediction so that ABC-P based on $s^{(1)}$ produces reasonable approximations.

#### Growing queue

We use $\theta _{\text {true}}=(8, 16, 0.15)$ in our second example so that the arrivals are relatively frequent as compared to the service times. The queue is mostly full in this case and the interarrival times $y_i$ have approximately the same distribution as the service times $u_i$. We observe that $\omega _i-\omega _{i-1} = y_i-w_i$ then approximately follows the same distribution as $u_i-w_i$. That is, the increments of the process $\{\omega _i\}_{i\ge 1}$ tend to be i.i.d. distributed so that $\{\omega _i\}_{i\ge 1}$ is approximately a random walk. Furthermore, as $u_i-w_i$ tends to be positive, the waiting times approximately grow linearly in expectation. The data set used is shown in Fig. 2.

**Fig. 4:**
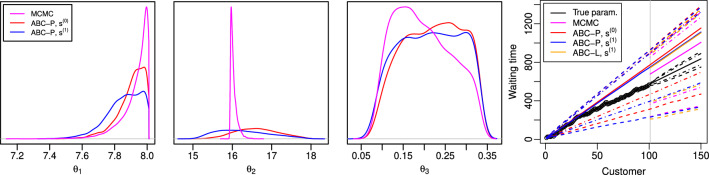
Typical results for the M/G/1 experiment of Sect. 5.1.3. *The first three plots* on the left show the marginal posteriors for $\theta$. *The fourth plot* illustrates the predictive densities for $\omega$ (solid lines: the median, dot-dashed lines: 75% credible interval (CI), dashed lines: 90% CI). Note that these lines are also drawn for the observed customers in the case of ABC-P. The vertical gray line shows the first future customer. The black rings show the unobserved waiting times not used for inference

Figure 4 shows that the marginal ABC posterior distributions of $\theta _1$ and $\theta _2$ feature similar trends as before while those of $\theta _3$ are equally good this time. The posterior uncertainty of $\theta _3$ is however noticeable because the observed interdeparture times are essentially determined by the service times that depend only on $\theta _1$ and $\theta _2$. Similarly, the observations are not very informative for estimating future waiting times and the ideal predictive density substantially benefits from its use of the true values of $\theta$ and $v$. Figure 4 further shows that the waiting times are growing approximately linearly as the above analysis suggests. All ABC approaches produce similar predictive densities which are comparable to the ground-truth. It is intuitive that the knowledge of the order of the interdeparture times or the additional summaries in $s^{(1)}$ are not highly useful when the queue is growing fairly steadily.

### Stochastic Lotka–Volterra model

We consider the Lotka–Volterra model which is a Markov jump process that describes the continuous-time evolution of a population of predators interacting with a prey population. This model also serves as a basic example of stochastic kinetics networks used to model the evolution of interacting molecules, see e.g. Wilkinson (2019). The size of the prey population at time *t* is $y^{ 1 }_t$ and similarly $y^{ 2 }_t$ is the predator population. We also denote $y_t ~{:}{=}~ (y^{ 1 }_t,y^{ 2 }_t)$. The model dynamics are Markovian with the transition probabilities

36 $$\begin{aligned}{} & {} \mathbb {P}(y_{t+\Delta t}=(w_1',w_2') \,|\,y_{t}=(w_1,w_2), \theta ) \nonumber \\{} & {} \quad = {\left\{ \begin{array}{ll} 1-\gamma _{\theta }(w)\Delta t+ o(\Delta t), &{} \text {if } w_1'=w_1, w_2'=w_2, \\ \theta _1 w_1\Delta t+ o(\Delta t), &{} \text {if } w_1'=w_1+1, w_2'=w_2, \\ \theta _2 w_1 w_2\Delta t+ o(\Delta t), &{} \text {if } w_1'=w_1-1, w_2'=w_2+1, \\ \theta _3 w_2\Delta t+ o(\Delta t), &{} \text {if } w_1'=w_1, w_2'=w_2-1, \\ o(\Delta t), &{} \text {otherwise}, \end{array}\right. } \end{aligned}$$

where $\gamma _{\theta }(w) ~{:}{=}~ \theta _1 w_1 + \theta _2 w_1 w_2 + \theta _3 w_2$, $\Delta t$ is a small time increment and $\theta = (\theta _1,\theta _2,\theta _3)$ are positive rate parameters.

While the likelihood function associated with this model is intractable (except for some special scenarios), realizations can be simulated given any initial state and parameters $\theta$ using the Gillespie algorithm (Gillespie 1977). The Markov property implies that one can also simulate from $\pi (\tilde{y}\,|\,y,\theta ) = \pi (\tilde{y}\,|\,y_{\tau },\theta )$ where $\tilde{y}$ represent the population sizes at some future time points, *y* the observed populations and $y_{\tau }$ the observed populations at the last observation time $\tau$. However, if e.g. only the prey population is observed then ABC-F approach cannot be directly implemented via Gillespie algorithm because $y_{\tau }$ is not fully known. Particle MCMC methods can be in principle used for inference in the noisy case, see Wilkinson (2019), Warne et al. (2020) and references therein for details. In this paper we however limit our attention to ABC methods in the non-noisy setting.

#### Common experimental details

The following settings are common to all Lotka–Volterra experiments considered: The initial state is $y_0=(100,50)$ and we use $\theta _{\text {true}}=(1, 0.005, 0.6)$ which produces oscillating behavior of the populations. The parameters are log-transformed for inference and we use the prior $(\log \theta _1,\log \theta _2,\log \theta _3) \sim {{\,\mathrm{\mathcal {U}}\,}}([-6,2]^3)$. We use similar strategies for forming the summary statistics, selecting thresholds and the same settings for the ABC-MCMC sampler as in the M/G/1 experiments. In particular, the acceptance probability of ABC-MCMC is $2\%$ and $2\cdot 10^6$ iterations are run. We use $L^{\infty }$-norm for $||\cdot ||'$ but this time $||\cdot ||$ is the weighted Euclidean distance whose weights are reciprocals of the squared median absolute deviations (MAD) of the simulated summaries estimated empirically as before. Summary statistics are designed individually for each inference task as explained below.

#### Prediction tasks

We consider a prediction task where the population sizes at 81 equidistant times in $[0,{\tau _{1}}]$ are observed and the main goal is to compute the posterior predictive distribution of both populations in $[{\tau _{1}},{\tau _{2}}]$. We select ${\tau _{1}}=24$ and ${\tau _{2}}=45$ time units. We study two cases: 1) Both populations are observed and we compare ABC-P and ABC-F and 2) Only prey populations $y^{ 1 }_t$ are observed and we compare ABC-P and ABC-L. ABC-L is implemented by setting $v=y^{ 2 }_{{\tau _{1}}}$ in case 2.

Similarly to earlier analyses (e.g. Papamakarios and Murray 2016; Wilkinson 2019) where the goal is to infer $\theta$, we use the following summary statistics for our case 1: the mean, standard deviation, two first autocorrelations of both populations and cross-correlation between both populations. The resulting 9-dimensional baseline summary statistic is denoted by $s^{(0)}$. We then form a summary statistic $s^{(1)}$ specifically for the prediction task at hand: We set $\bar{s}^{(1)} = s^{(0)}$ and $\tilde{s}(y) = y_{{\tau _{1}}}\in \mathbb {R}^2$. The baseline summary statistic $s^{(0)}$ for case 2 includes the mean, standard deviation and two first autocorrelations of the prey population. Summary statistic $s^{(1)}$ is formed so that $\bar{s}^{(1)} = s^{(0)}$ and^7^$\tilde{s}(y) = (y^{ 1 }_{{\tau _{1}}-i\delta })_{i=0}^{5}\in \mathbb {R}^{6}$ where $\delta =0.3$ is the time between consecutive observations. We use $\tilde{h}=50$ in case 1 and $\tilde{h}=30$ in case 2. In case 1 we compare our ABC methods to the ideal predictive distribution $\pi (\tilde{y}\,|\,y, \theta _{\text {true}})=\pi (\tilde{y}\,|\,y_{{\tau _{1}}}, \theta _{\text {true}})$ from which we directly simulate using the Gillespie algorithm. In case 2 our comparison is to an approximation of $\pi (\tilde{y}\,|\,y^{ 1 }, \theta _{\text {true}})$ which is obtained by first generating $(\tilde{y}^{(i)},z^{(i)}) \sim \pi (\tilde{y},z\,|\,\theta _{\text {true}}), i=1,\ldots ,10^6$, using the Gillespie algorithm and accepting $\tilde{y}^{(i)}$ if $||\tilde{s}(z^{(i)})-\tilde{s}(y)||'=\max _{i\in \{0,\ldots ,5\}}|z^{\smash { 1 (i)}}_{{\tau _{1}}-i\delta }-y^{ 1 }_{{\tau _{1}}-i\delta }|\le 7$. This is a form of ABC rejection sampling (but conditionally on $\theta _{\text {true}}$, see Supplementary material A for discussion).

**Fig. 5:**
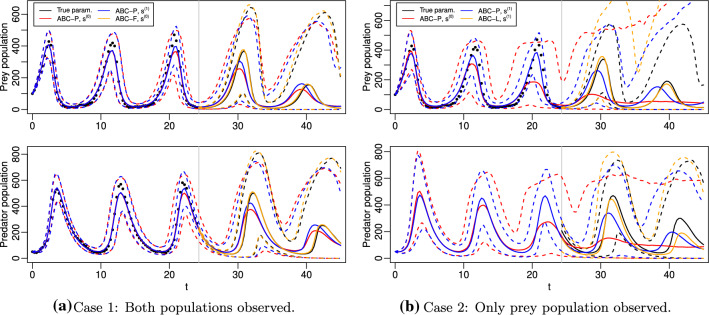
Typical results for the Lotka–Volterra experiments of Sect. 5.2.2. The vertical gray line shows where the prediction interval starts and the black circles show the observations. The solid lines show the median and the dashed lines the $90\%$ CI. These lines are also drawn in $[0,{\tau _{1}}]$ to demonstrate that the observed data is not conditioned on exactly in ABC. “True param.” shows the ideal predictive distribution based on $\theta _{\text {true}}$ (case 1) and its approximation (case 2). The prey population size is truncated in case 2 because the predator population can die out with a non-negligible posterior probability in which case the prey population starts to grow exponentially fast

Typical results for case 1 are shown in Fig. 5a. All ABC posterior predictive densities roughly capture the future model dynamics. The mean absolute error of the posterior median prediction (abbreviated as MAE and where the mean is taken over both populations and the prediction interval) of ABC-P is 40 with both $s^{(0)}$ and $s^{(1)}$. Statistic $s^{(1)}$ however produces smaller standard deviation (std) of the posterior predictive in the region $t\approx {\tau _{1}}$ than $s^{(0)}$ (e.g. 27 vs. 70 for the predator population at $t={\tau _{1}}+\delta$). While both approaches produce visibly inflated $90\%$ CI there, the accuracy with $s^{(1)}$ could be increased at the cost of more simulations by lowering $\tilde{h}$ as this quantity directly controls the absolute error at $t={\tau _{1}}$. However, we do not have such apparent guarantee with $s^{(0)}$. The ABC-F posterior predictive is similar to the ideal predictive density over the whole interval $[{\tau _{1}},{\tau _{2}}]$ (MAE is 5.3 and the std of the posterior predictive of the predator population at $t={\tau _{1}}+\delta$ is 6.6 while that of the ideal baseline is 6.4) so that ABC-F is clearly preferable to ABC-P.

**Fig. 6:**
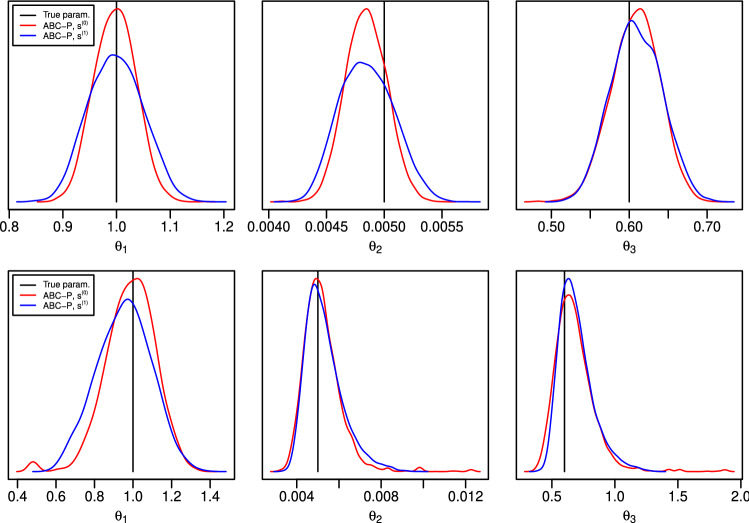
Posterior distributions for the parameters of the Lotka–Volterra experiments corresponding to Fig. 5 and Sect. 5.2.2. *Top row:* Case 1 where both populations are observed. *Bottom row:* Case 2 where only prey population is observed. The black vertical line shows the true value of the parameter

Figure 5b shows that, unsurprisingly, prediction in case 2 is more challenging than in case 1. ABC-P based on $s^{(1)}$ produces visibly more accurate posterior predictive than $s^{(0)}$. MAE for ABC-P with $s^{(0)}$ is 72 and with $s^{(1)}$ it is 54. ABC-L produces the most accurate median prediction (MAE is 24) and also the least inflated posterior predictive of the prey population in the region $t\approx {\tau _{1}}$ as a consequence of its ability to partially use the exact value of the observation $y^{ 1 }_{{\tau _{1}}}$. ABC-P based on $s^{(1)}$ and ABC-L however both produce similar predictive posteriors for the unobserved predator population $y^{ 2 }_{t}$ at $t\approx {\tau _{1}}$. Marginal ABC posterior distributions for $\theta$ in both cases are finally shown Fig. 6. Summary statistic $s^{(1)}$ produces slightly more inflated posterior as $s^{(0)}$ similarly—and for the same reason—as in the M/G/1 experiments.

#### Missing data task

The goal here is to estimate the unobserved population sizes in $[{\tau _{1}},{\tau _{2}}]$ based on 51 equidistant observations both in $[0,{\tau _{1}}]$ and $[{\tau _{2}},{\tau _{3}}]$ with $0<{\tau _{1}}<{\tau _{2}}<{\tau _{3}}$. In particular, we use ${\tau _{1}}=15, {\tau _{2}}=36$ and ${\tau _{3}}=51$. The summary statistics of Sect. 5.2.2 are not meaningful and we instead use the mean and standard deviation of both population sizes separately computed for intervals $[0,{\tau _{1}}]$ and $[{\tau _{2}},{\tau _{3}}]$. This produces 8-dimensional summary statistic $\bar{s}^{(1)}$. Additionally, we choose $\tilde{s}^{(1)}(y) = (y_{{\tau _{1}}},y_{{\tau _{2}}})\in \mathbb {R}^4$ and $\tilde{h}=50$. Only ABC-P is considered as the other ABC approaches do not to apply^8^. We compare it to the ideal predictive distribution $\pi (\tilde{y}\,|\,y, \theta _{\text {true}})=\pi (\tilde{y}\,|\,y_{{\tau _{1}}}, y_{{\tau _{2}}}, \theta _{\text {true}})$ which is computed in a similar fashion as the one in case 2 of Sect. 5.2.2: We first generate $10^6$ samples $(\tilde{y}^{(i)},z_{{\tau _{2}}}^{\smash {(i)}}) \sim \pi (\tilde{y},z_{{\tau _{2}}}\,|\,y_{{\tau _{1}}}, \theta _{\text {true}})$ and then accept those of them that satisfy $||z_{{\tau _{2}}}^{\smash {(i)}}-y_{{\tau _{2}}}||'\le 5$ (our first data set) or that match the observed $y_{{\tau _{2}}}$ exactly (our second data set). In the latter case we obtain exact samples from $\pi (\tilde{y}\,|\,y_{{\tau _{1}}}, y_{{\tau _{2}}}, \theta _{\text {true}})$.

**Fig. 7:**
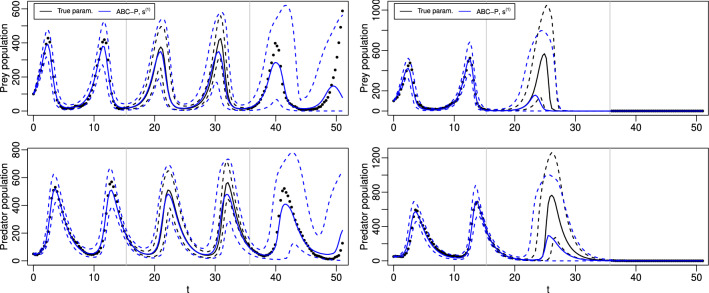
Results for the Lotka–Volterra experiment of Sect. 5.2.3 with two sets of observed data. The vertical gray lines indicate the unobserved time interval [15, 36] and the black circles show the observations. The solid lines show the median and the dashed lines the $90\%$ CI. “True param.” shows the ideal predictive distribution based on $\theta _{\text {true}}$ (right column) and its approximation (left column)

Results with two data realizations are shown in Fig. 7. In a typical case shown in the left column, the approximation is good as compared to the approximate ideal predictive density (MAE is 17). As before, the uncertainty is overestimated especially near ${\tau _{1}}$ and ${\tau _{2}}$ as the matching to the observed $y_{{\tau _{1}}}$ and $y_{{\tau _{2}}}$ is only done up to the threshold $\tilde{h}$. The right column presents a special situation where both populations have become extinct. The approximation quality is fair in this case (MAE is 59). The ABC posteriors for $\theta$ are additionally shown in Supplementary material C.2.

## Discussion

There is no single natural, general way to to approximate the posterior predictive distribution of intractable models via ABC. In this paper we have studied three such complementary approaches summarized in Table 1. These methods make different assumptions regarding which predictive density of the model can be simulated from. We provided some new insight to the earlier approach, abbreviated as ABC-F in this paper, and pointed out its limitations. We then proposed related but more general ABC-P and ABC-L methods, investigated their basic properties (especially the selection of summary statistics), discussed some ways to implement these methods in practice, and finally provided numerical examples of all three methods.

Our analysis indicates that forming summary statistics for ABC-P and weighting them appropriately requires more care than for the standard ABC inference or for ABC-F because these statistics need to be informative in the sense of predictive sufficiency instead of ordinary sufficiency. Of course, they should also be low-dimensional to avoid the curse of dimensionality. Such suitable statistics may have large variability which can lead to more pronounced computational challenges than in standard ABC inference and in some situations result substantially inflated predictive intervals. Also, suitable low-dimensional statistics may not exist which could be the case e.g. with intractable spatial data models which were however not analyzed in this paper. On the other hand, such suitable statistics can be often constructed when the model features Markovian or some other simplifying structure. ABC-P produced reasonable posterior predictive approximations in our experiments although we used a basic ABC-MCMC implementation and summary statistics designed for prediction in a rather rudimentary fashion.

Our analysis and empirical results suggest that in general ABC-F should be used instead of ABC-P. However, as we have discussed, implementing ABC-F can be difficult or infeasible which makes ABC-P and ABC-L a useful addition to statistician’s toolbox. We cannot conclude whether ABC-L should be preferred to ABC-P when both approaches are feasible. This likely depends on the model at hand and the suitability of the selected summary statistics which is often hard to assess in practice. It should also be noted that when asymptotically exact (particle) MCMC or other model-specific methods can be used, they are likely more accurate than the more generic, approximate methods analyzed in this paper.

Our key aim was to give a unified overview on approximating the posterior predictive via ABC in an accessible manner. As future work, one could study more closely how to best use such methods for specific models and inference tasks. One could also investigate if LFI techniques such as regression adjustment (Beaumont et al. 2002; Blum 2010), Bayesian synthetic likelihood (Price et al. 2018), ratio density estimation (Thomas et al. 2022) or conditional density estimation methods based on neural networks or Gaussian processes (Papamakarios and Murray 2016; Papamakarios et al. 2019; Grazian and Fan 2020; Järvenpää et al. 2020) can be used to improve the accuracy or computational efficiency of the ABC-P approach. Another important research direction would be to study how to construct informative summary statistics for predictive ABC inference in an automatic fashion. Such methods have been developed for standard ABC inference (see e.g. Sisson et al. (2019, Chapter 5)) and some of them could possibly be extended to the predictive setting. Also, our analysis on the summary statistics for prediction might offer new perspective for selecting them for standard ABC inference.

## Supplementary Information

Below is the link to the electronic supplementary material.

Supplementary file 1 (pdf 462 KB)

## Footnotes

1 The function *s* can depend also on *x* (and observed data *y* even when computed for pseudo-data *z*) although this is not explicitly shown in our notation for brevity.

2 Other LFI posterior approximations can also be used and $\pi (\tilde{y}\,|\,\theta ,y)$ can be replaced with $\pi (\tilde{y}\,|\,\theta ,s'_y)$ where $s'_y$ is some summary statistic. Sampling from $\pi (\tilde{y}\,|\,\theta ,s'_y)$ can however be difficult even if sampling from $\pi (\tilde{y}\,|\,\theta ,y)$ is feasible (and vice versa).

3 This character may also refer to Frazier et al. (2019) who analyzed this approach and used the term *forecasting* while we (interchangeably) use *prediction* in this paper.

4 A technical subtlety is that one could require the condition $\pi (\theta \,|\,s_y) = \pi (\theta \,|\,y)$ to hold only for the prior density actually used for ABC inference instead of all possible choices as in the definition of Bayes sufficiency. In the following analysis we do not need to take a stand which form should hold.

5 Deriving an analytical formula for $\widehat{\pi }^{\,\smash {\text {(P)}}}_{h}(y_{n+1} \,|\,y_n, \bar{y}_{\phi })$ is difficult and this quantity presumably has no simple formula when $h>0$ so we consider only the $h=0$ case.

6 Since the model is neither black-box nor highly complex, it might be possible to devise an algorithm to sample from $\pi (\tilde{\omega }\,|\,y, \theta )$. Whether such algorithm could be used for this particular case is however irrelevant for our aims here.

7 While $y_{{\tau _{1}}}$ is sufficient in the sense of (13) in case 1 due to the Markov property, this does not hold for $y^{ 1 }_{{\tau _{1}}}$ when only the prey is observed. Due to this fact and guided by preliminary experiments we include additional observations to $\tilde{s}$.

8 Special methods called bridges have been proposed to approximately draw from $\pi (\tilde{y}\,|\,y_{{\tau _{1}}}, y_{{\tau _{2}}}, \theta )$ in the case of some Markov jump processes, see Golightly and Sherlock (2019). It seems possible to combine them with ABC-F but we do not analyze the feasibility of such model-specific approach in this paper.
