# Supplementary material for: On predictive inference for intractable models via approximate Bayesian computation
Source: Stat Comput. 2023 Feb 9;33(2):42. doi: 10.1007/s11222-022-10163-6 (PMC9911513; doi:10.1007/s11222-022-10163-6)
Supplement: Supplementary file 1 — (pdf 462 KB) [file 11222_2022_10163_MOESM1_ESM.pdf]

Supplementary materials for "On predictive inference for intractable models via approximate Bayesian computation"

Marko Järvenpää, Jukka Corander

October 14, 2022

A An alternative to ABC-P

ABC-P is based on approximating $\pi(\tilde{y}, y \,|\, \theta)$. One could also approximate $\pi(\tilde{y} \,|\, \theta, y)$ with

$$\widehat{\pi}_{\tilde{h}}(\tilde{y} \,|\, \theta, \tilde{s}_y) := \frac{\int \tilde{K}_{\tilde{h}}(\tilde{\Delta}(\tilde{s}_y, \tilde{s}_{z'}))\pi(\tilde{y}, \tilde{s}_{z'} \,|\, \theta)\,\mathrm{d}\tilde{s}_{z'}}{\iint \tilde{K}_{\tilde{h}}(\tilde{\Delta}(\tilde{s}_y, \tilde{s}_{z'}))\pi(\tilde{y}, \tilde{s}_{z'} \,|\, \theta)\,\mathrm{d}\tilde{s}_{z'}\,\mathrm{d}\tilde{y}} \tag{A.1}$$

for each θ. (A.1) is formed as the "standard" ABC posterior $\widehat{\pi}_h(\theta \,|\, s_y)$ except that $\tilde{y}$ appears in the place of θ and the parameter θ is conditioned on. This approximation can be useful when an accurate point estimate for θ is already available. This approximation is also used in Section 5.2, given the specific parameter value of $\theta = \theta_{\text{true}}$. The resulting approximate density is in fact exact (up to the sampling error) in the second data realization case of Section 5.2.3.

An alternative to ABC-P, which also requires only the ability to jointly sample from $\pi(\tilde{y}, y \,|\, \theta)$, is obtained by using (A.1) in the ABC-F definition (6) so that

$$\pi(\tilde{y} \,|\, y) \approx \widehat{\pi}^{(\text{P'})}_{\tilde{h},h}(\tilde{y} \,|\, \tilde{s}_y; s_y) := \int \widehat{\pi}_{\tilde{h}}(\tilde{y} \,|\, \theta, \tilde{s}_y)\widehat{\pi}_h(\theta \,|\, s_y)\,\mathrm{d}\theta. \tag{A.2}$$

We can also define the joint density[A.1]

$$\pi(\tilde{y}, \theta \,|\, y) \approx \widehat{\pi}^{(\text{P'})}_{\tilde{h},h}(\tilde{y}, \theta \,|\, \tilde{s}_y; s_y) := \widehat{\pi}_{\tilde{h}}(\tilde{y} \,|\, \theta, \tilde{s}_y)\widehat{\pi}_h(\theta \,|\, s_y). \tag{A.3}$$

This approach is referred as ABC-P'. Note that a different threshold $\tilde{h}$, kernel $\tilde{K}$, summary statistic $\tilde{s}$ and discrepancy $\tilde{\Delta}$ can be used for the "nested" ABC approximation $\widehat{\pi}_{\tilde{h}}(\tilde{y} \,|\, \theta, \tilde{s}_y)$ as for $\widehat{\pi}_h(\theta \,|\, s_y)$. The approximation $\widehat{\pi}_{\tilde{h}}(\tilde{y} \,|\, \theta, \tilde{s}_y)$ uses a separate set of pseudo-data which we have denoted by z' to distinguish it from z used in $\widehat{\pi}_h(\theta \,|\, s_y)$.

We can write the ABC-P' approximation as

$$\begin{aligned}
\widehat{\pi}^{(\text{P'})}_{\tilde{h},h}(\tilde{y}, \theta \,|\, \tilde{s}_y; s_y) &= \widehat{\pi}_{\tilde{h}}(\tilde{y} \,|\, \theta, \tilde{s}_y)\widehat{\pi}_h(\theta \,|\, s_y) && \text{(A.4)}\\
&= \frac{\int \tilde{K}_{\tilde{h}}(\tilde{\Delta}(\tilde{s}_y, \tilde{s}_{z'}))\pi(\tilde{y}, \tilde{s}_{z'} \,|\, \theta)\,\mathrm{d}\tilde{s}_{z'}}{\iint \tilde{K}_{\tilde{h}}(\tilde{\Delta}(\tilde{s}_y, \tilde{s}_{z'}))\pi(\tilde{y}, \tilde{s}_{z'} \,|\, \theta)\,\mathrm{d}\tilde{s}_{z'}\,\mathrm{d}\tilde{y}} \frac{\int K_h(\Delta(s_y, s_z))\pi(s_z \,|\, \theta)\pi(\theta)\,\mathrm{d}s_z}{\iint K_h(\Delta(s_y, s_z))\pi(s_z \,|\, \theta)\pi(\theta)\,\mathrm{d}s_z\,\mathrm{d}\theta} && \text{(A.5)}\\
&= \frac{\int \tilde{K}_{\tilde{h}}(\tilde{\Delta}(\tilde{s}_y, \tilde{s}_{z'}))\pi(\tilde{y}, \tilde{s}_{z'} \,|\, \theta)\pi(\theta)\,\mathrm{d}\tilde{s}_{z'}}{\iiint K_h(\Delta(s_y, s_z))\pi(\tilde{y}, s_z \,|\, \theta)\pi(\theta)\,\mathrm{d}s_z\,\mathrm{d}\theta\,\mathrm{d}\tilde{y}} \frac{\widehat{\pi}_h(s_y \,|\, \theta)}{\widehat{\pi}_{\tilde{h}}(\tilde{s}_y \,|\, \theta)}, && \text{(A.6)}
\end{aligned}$$

[A.1] We of course cannot directly manipulate this joint density using the basic fact $\pi(a, b) = \pi(a \,|\, b)\pi(b) = \pi(b \,|\, a)\pi(a)$ (where a and b are random vectors) since $\widehat{\pi}_{\tilde{h}}(\tilde{y} \,|\, \theta, \tilde{s}_y)$ and $\widehat{\pi}_h(\theta \,|\, s_y)$ are conditioned on different summary statistics. Similar observation also holds for ABC-F.

where $\widehat{\pi}_h(s_y \mid \theta)$ and $\widehat{\pi}_{\tilde{h}}(\tilde{s}_y \mid \theta)$ denote ABC likelihoods, see Section 2.2. Now, if $\tilde{s} = s$, $\tilde{h} = h$, $\tilde{K} = K$ and $\tilde{\Delta} = \Delta$, then $\widehat{\pi}_h(s_y \mid \theta) = \widehat{\pi}_{\tilde{h}}(\tilde{s}_y \mid \theta)$ so that these terms cancel out in (A.6) and the resulting formula equals (9). This shows that ABC-P' defines the same target approximation as ABC-P in this particular case. Hence, ABC-P' could be considered as a generalization of ABC-P.

ABC-P' does not seem to provide evident, general advantages over its special case ABC-P. For example, matching of predictive sufficient summary statistics is required in both cases. Specifically, $\tilde{s}$ needs to ideally satisfy the condition (13) while s needs to be parametric sufficient satisfying (12). Also, it is not immediately clear how to most efficiently sample from (A.2) as the normalization constant of $\widehat{\pi}_{\tilde{h}}(\tilde{y} \mid \theta, \tilde{s}_y)$ depends on θ or how to determine $\tilde{h}$ which in principle could depend on θ. On the other hand, ABC-P' might facilitate more convenient implementation than ABC-P in a sense that the estimation of θ and $\tilde{y}$ can be separated. More detailed investigation is however left as a potential topic for future work.

B Additional mathematical details

B.1 Proof of Proposition 3.1

We obtain

$$
\begin{aligned}
\lim_{t\to\infty} \widehat{\pi}_{h_t}^{(\mathrm{P})}(\tilde{y} \mid s_y) &= \lim_{t\to\infty} \frac{\iint \mathbb{1}_{s_z \in \mathcal{A}_t} \pi(\tilde{y}, s_z \mid \theta)\pi(\theta)\, \mathrm{d}s_z\, \mathrm{d}\theta}{\iiint \mathbb{1}_{s_z \in \mathcal{A}_t} \pi(\tilde{y}, s_z \mid \theta)\pi(\theta)\, \mathrm{d}s_z\, \mathrm{d}\theta\, \mathrm{d}\tilde{y}} && \text{(B.7)}\\
&= \lim_{t\to\infty} \frac{\iint \mathbb{1}_{s_z \in \mathcal{A}_t} \pi(\tilde{y}, s_z, \theta)\, \mathrm{d}\theta\, \mathrm{d}s_z}{\iiint \mathbb{1}_{s_z \in \mathcal{A}_t} \pi(\tilde{y}, s_z, \theta)\, \mathrm{d}\theta\, \mathrm{d}\tilde{y}\, \mathrm{d}s_z} && \text{(B.8)}\\
&= \lim_{t\to\infty} \frac{\int_{s_z \in \mathcal{A}_t} \pi(\tilde{y}, s_z)\, \mathrm{d}s_z}{\int_{s_z \in \mathcal{A}_t} \pi(s_z)\, \mathrm{d}s_z} && \text{(B.9)}\\
&= \frac{\lim_{t\to\infty} \frac{1}{|\mathcal{A}_t|} \int_{s_z \in \mathcal{A}_t} \pi(\tilde{y}, s_z)\, \mathrm{d}s_z}{\lim_{t\to\infty} \frac{1}{|\mathcal{A}_t|} \int_{s_z \in \mathcal{A}_t} \pi(s_z)\, \mathrm{d}s_z} && \text{(B.10)}\\
&= \frac{\pi(\tilde{y}, s_y)}{\pi(s_y)} && \text{(B.11)}\\
&= \pi(\tilde{y} \mid s_y), && \text{(B.12)}
\end{aligned}
$$

where on the second line we have used Tonelli's theorem to change the order of integration and where the fifth equality holds almost everywhere and follows from Lebesgue differentiation theorem (see e.g. Rudin (1987, Chapter 7) or Stein and Shakarchi (2005, Chapter 3)) which requires (C4) and (C5).

Similar reasoning as above further shows that

$$
\begin{aligned}
\lim_{t\to\infty} \widehat{\pi}_{h_t}^{(\mathrm{P})}(\tilde{y}, \theta \mid s_y) &= \lim_{t\to\infty} \frac{\int \mathbb{1}_{s_z \in \mathcal{A}_t} \pi(\tilde{y}, s_z \mid \theta)\pi(\theta)\, \mathrm{d}s_z}{\iiint \mathbb{1}_{s_z \in \mathcal{A}_t} \pi(\tilde{y}, s_z \mid \theta)\pi(\theta)\, \mathrm{d}s_z\, \mathrm{d}\theta\, \mathrm{d}\tilde{y}} && \text{(B.13)}\\
&= \lim_{t\to\infty} \frac{\int_{s_z \in \mathcal{A}_t} \pi(\tilde{y}, \theta, s_z)\, \mathrm{d}s_z}{\int_{s_z \in \mathcal{A}_t} \pi(s_z)\, \mathrm{d}s_z} && \text{(B.14)}\\
&= \frac{\lim_{t\to\infty} \frac{1}{|\mathcal{A}_t|} \int_{s_z \in \mathcal{A}_t} \pi(\tilde{y}, \theta, s_z)\, \mathrm{d}s_z}{\lim_{t\to\infty} \frac{1}{|\mathcal{A}_t|} \int_{s_z \in \mathcal{A}_t} \pi(s_z)\, \mathrm{d}s_z} && \text{(B.15)}\\
&= \frac{\pi(\tilde{y}, \theta, s_y)}{\pi(s_y)} && \text{(B.16)}\\
&= \pi(\tilde{y}, \theta \mid s_y), && \text{(B.17)}
\end{aligned}
$$

where the fourth equality holds almost everywhere as in the previous case.

B.2 Mathematical details related to Example 3.1

We justify the various results in Example 3.1. Filling the details of some straightforward computations are left for the reader. First, we compute

$$\pi(c\,|\,y,\phi,\sigma^2) \propto \prod_{i=1}^{n} \pi(y_i\,|\,y_{i-1},c,\phi,\sigma^2)\pi(c) \propto \prod_{i=1}^{n} \mathcal{N}(y_i\,|\,c+\phi y_{i-1},\sigma^2) \tag{B.18}$$

$$\propto e^{-\frac{1}{2\sigma^2}(y_1-c)^2} \prod_{i=2}^{n} e^{-\frac{1}{2\sigma^2}(x_i-c-\phi y_{i-1})^2} \propto e^{-\frac{1}{2\sigma^2}[nc^2-2c(\sum_{i=1}^{n} y_i)-\phi\sum_{i=1}^{n-1} y_i]} \tag{B.19}$$

$$\propto \mathcal{N}(c\,|\,\bar{y}_\phi,\sigma^2/n), \tag{B.20}$$

which shows $\bar{y}_\phi$ is parametric sufficient. Since $\pi(y_{n+p}\,|\,y_{1:n},\theta) = \pi(y_{n+p}\,|\,y_n,\theta)$ for any $p \geq 1$ by the Markov property, $(\bar{y}_\phi, y_n)$ is predictive sufficient for any y_{n+p}, and in particular for y_{n+1}.

In the rest of this section[B.2] we assume $|\phi| < 1$. First we notice that the Markov process can be written as

$$y_t = c\frac{1-\phi^t}{1-\phi} + \sum_{i=1}^{t} \phi^{t-i}\varepsilon_i, \quad t = 0,1,\ldots \tag{B.21}$$

where we have used the geometric sum formula which holds because $\phi \neq 1$. Using (B.21), the basic properties of expectation and (co)variance, and the fact that ε_i are i.i.d., one can show that[B.3]

$$\mathbb{E}(y_t\,|\,\theta) = c\frac{1-\phi^t}{1-\phi}, \tag{B.22}$$

$$\mathbb{V}(y_t\,|\,\theta) = \sigma^2\frac{1-\phi^{2t}}{1-\phi^2}, \tag{B.23}$$

$$\mathrm{cov}(y_s,y_t\,|\,\theta) = \sigma^2\phi^{|s-t|}\frac{1-\phi^{2\min\{s,t\}}}{1-\phi^2} \tag{B.24}$$

for any $s,t = 1,2,\ldots$. Recall that $\bar{y}_\phi := ((1-\phi)\sum_{i=1}^{n-1} y_i + y_n)/n$. Since $y_{1:n}$ are clearly jointly Gaussian and since $\bar{y}_\phi$ depends linearly on $y_{1:n}$, $\bar{y}_\phi$ also follows Gaussian distribution whose mean and variance can be easily shown to be

$$\mathbb{E}(\bar{y}_\phi\,|\,\theta) = c, \tag{B.25}$$

$$\mathbb{V}(\bar{y}_\phi\,|\,\theta) = \sigma^2/n. \tag{B.26}$$

Next, using (B.25), (B.26) and a standard Gaussian identity, we obtain (16):

$$\widehat{\pi}_h(c\,|\,\bar{y}_\phi) \propto \int_{-\infty}^{\infty} \mathcal{N}(\bar{y}_\phi\,|\,\bar{z}_\phi,h^2)\mathcal{N}(\bar{z}_\phi\,|\,c,\sigma^2/n)\pi(c)\,\mathrm{d}\bar{z}_\phi \propto \mathcal{N}(c\,|\,\bar{y}_\phi,\sigma^2/n+h^2). \tag{B.27}$$

Using (B.22), (B.23) and (B.24) we obtain

$$\begin{bmatrix} y_{n+p} \\ y_n \end{bmatrix} \Bigg|\, \theta \sim \mathcal{N}_2\left(\frac{c}{1-\phi}\begin{bmatrix} 1-\phi^{n+p} \\ 1-\phi^n \end{bmatrix}, \frac{\sigma^2}{1-\phi^2}\begin{bmatrix} 1-\phi^{2(n+p)} & \phi^p(1-\phi^{2n}) \\ \phi^p(1-\phi^{2n}) & 1-\phi^{2n} \end{bmatrix}\right). \tag{B.28}$$

Using a well-known formula for conditional Gaussian density further produces

$$y_{n+p}\,|\,y_n,\theta \sim \mathcal{N}\left(c\frac{1-\phi^p}{1-\phi} + \phi^p y_n, \sigma^2\frac{1-\phi^{2p}}{1-\phi^2}\right). \tag{B.29}$$

[B.2] The following results in fact hold more generally, e.g. (B.22) when $\phi \neq 1$ and (B.23) when $|\phi| \neq 1$.

[B.3] When $|\phi| < 1$ and if we let $t \to \infty$, we obtain the well-known formulas for the mean and variance of the stationary order one autoregressive model AR(1).

Using $\pi(y_{n+p}\,|\,y_{1:n},\theta) = \pi(y_{n+p}\,|\,y_n,\theta)$, (B.29) and (B.27) we obtain

$$\widehat{\pi}_h^{(\mathrm{F})}(y_{n+p}\,|\,y;\bar{y}_\phi) = \int_{-\infty}^{\infty} \pi(y_{n+p}\,|\,y,c)\widehat{\pi}_h(c\,|\,\bar{y}_\phi)\,\mathrm{d}c \tag{B.30}$$

$$= \mathcal{N}\bigg(y_{n+p}\,\bigg|\,\mathring{y}_{\phi,p}, \sigma^2\frac{1-\phi^{2p}}{1-\phi^2} + \frac{(1-\phi^p)^2}{(1-\phi)^2}(\sigma^2/n+h^2)\bigg), \quad \mathring{y}_{\phi,p} := \frac{1-\phi^p}{1-\phi}\bar{y}_\phi + \phi^p y_n, \tag{B.31}$$

where the integral is computed by deducing that the result is Gaussian density whose mean (variance) follows by using the law of total expectation (variance) or, alternatively, by applying Gaussian identities. The result (21) follows immediately by setting $p=1$. Similarly, the discussion below (B.20) and the result (B.31) with $p=1$ and $h=0$ imply (17). As the resulting formula depends on data $y_{1:n}$ only via $\mathring{y}_\phi$, the summary statistic $\mathring{y}_\phi$ is predictive sufficient (in the weaker sense).

We then justify (18) and (20). As the latter result follows by similar computations but with $\mathring{y}_\phi$ in place of $\bar{y}_\phi$, we only outline the derivation of the former result. Using (B.24) and some straightforward computations we obtain

$$\mathrm{cov}(y_{n+1},\bar{y}_\phi\,|\,\theta) = \sigma^2\frac{\phi(1-\phi^n)}{n(1-\phi)}. \tag{B.32}$$

The joint distribution of $y_{1:n+1} = (y_1,\ldots,y_{n+1})$ is clearly Gaussian and so is that of $(y_{1:n+1},\bar{y}_\phi)$. By using (B.22), (B.23), (B.25), (B.26) and (B.32), we further obtain

$$\begin{bmatrix} y_{n+1} \\ \bar{y}_\phi \end{bmatrix}\,\Bigg|\,\theta \sim \mathcal{N}_2\left(c\begin{bmatrix}\frac{1-\phi^{n+1}}{1-\phi} \\ 1\end{bmatrix}, \sigma^2\begin{bmatrix}\frac{1-\phi^{2(n+1)}}{1-\phi^2} & \frac{\phi(1-\phi^n)}{n(1-\phi)} \\ \frac{\phi(1-\phi^n)}{n(1-\phi)} & \frac{1}{n}\end{bmatrix}\right). \tag{B.33}$$

This implies

$$\pi(y_{n+1}\,|\,\bar{y}_\phi,\theta) = \mathcal{N}\bigg(y_{n+1}\,\bigg|\,c + \frac{\bar{y}_\phi\phi(1-\phi^n)}{1-\phi}, \sigma^2\bigg(\frac{1-\phi^{2(n+1)}}{1-\phi^2} - \frac{\phi^2(1-\phi^n)^2}{n(1-\phi)^2}\bigg)\bigg). \tag{B.34}$$

Equation (18) then follows as

$$\widehat{\pi}_h^{(\mathrm{P})}(y_{n+1}\,|\,\bar{y}_\phi) \propto \iint \mathcal{N}(\bar{y}_\phi\,|\,\bar{z}_\phi,h^2)\pi(y_{n+1}\,|\,\bar{z}_\phi,c)\mathcal{N}(\bar{z}_\phi\,|\,c,\sigma^2/n)\pi(c)\,\mathrm{d}\bar{z}_\phi\,\mathrm{d}c \tag{B.35}$$

$$\propto \int \mathcal{N}(\bar{y}_\phi\,|\,\bar{z}_\phi,h^2)\int\mathcal{N}\bigg(y_{n+1}\,\bigg|\,c+\frac{\bar{y}_\phi\phi(1-\phi^n)}{1-\phi}, a_{\phi,n}\sigma^2\bigg)\mathcal{N}(c\,|\,\bar{z}_\phi,\sigma^2/n)\,\mathrm{d}c\,\mathrm{d}\bar{z}_\phi \tag{B.36}$$

$$= \int \mathcal{N}\big(y_{n+1}\,\big|\,b_{\phi,n}\bar{z}_\phi, a_{\phi,n}\sigma^2+\sigma^2/n\big)\,\mathcal{N}(\bar{z}_\phi\,|\,\bar{y}_\phi,h^2)\,\mathrm{d}\bar{z}_\phi \tag{B.37}$$

$$= \mathcal{N}\big(y_{n+1}\,\big|\,b_{\phi,n}\bar{y}_\phi, a_{\phi,n}\sigma^2+\sigma^2/n+b_{\phi,n}^2h^2\big), \tag{B.38}$$

where on the second line we used (B.34).

It remains to verify the inequality in (19):

$$a_{n,\phi}-1 = \frac{1-\phi^{2(n+1)}}{1-\phi^2} - \frac{\phi^2(1-\phi^n)^2}{n(1-\phi)^2} - 1 = \sum_{i=0}^{n}\phi^{2i} - \frac{\phi^2}{n}\bigg(\sum_{i=0}^{n-1}\phi^i\bigg)^2 - 1 \tag{B.39}$$

$$= \phi^2\left(\sum_{i=0}^{n-1}\phi^{2i} - \frac{1}{n}\bigg(\sum_{i=0}^{n-1}\phi^i\bigg)^2\right) \tag{B.40}$$

$$\geq 0, \tag{B.41}$$

where we have used the Cauchy-Schwarz inequality. Since (B.39) is a polynomial of order $2n$ there are at most n global minima where the equality holds (clearly $\phi=0$ and $\phi=1$ are such points) and elsewhere the inequality is strict.

B.3 Justification for Equation (22)

Suppose first $|\phi| > 1$. By using (B.22) and (B.23) we obtain

$$\mathbb{P}(|z_n - y_n| \leq \tilde{h} \,|\, c) = \frac{1}{\sqrt{2\pi\sigma^2}} \sqrt{\frac{1-\phi^2}{1-\phi^{2n}}} \underbrace{\int_{y_n - \tilde{h}}^{y_n + \tilde{h}} e^{-\frac{1}{2\sigma^2}\frac{1-\phi^2}{1-\phi^{2n}}\left(z_n - c\frac{1-\phi^n}{1-\phi}\right)^2} \,\mathrm{d}z_n}_{\leq 2\tilde{h}} \tag{B.42}$$

$$\leq \frac{\sqrt{2}\tilde{h}}{\sqrt{\pi\sigma^2}} \frac{\sqrt{\phi^2 - 1}}{\sqrt{\phi^{2n} - 1}}. \tag{B.43}$$

The maximum of (B.42) exists and is clearly obtained when $c\frac{1-\phi^n}{1-\phi} = y_n$ so that (B.43) gives its upper bound.

Suppose $|\phi| < 1$. The maximum wrt. c is again obtained when $c\frac{1-\phi^n}{1-\phi} = y_n$ and we then compute

$$\max_{c \in \mathbb{R}} \mathbb{P}(|z_n - y_n| \leq \tilde{h} \,|\, c) = \frac{1}{\sqrt{2\pi\sigma^2}} \sqrt{\frac{1-\phi^2}{1-\phi^{2n}}} \max_{c \in \mathbb{R}} \int_{y_n - \tilde{h}}^{y_n + \tilde{h}} e^{-\frac{1}{2\sigma^2}\frac{1-\phi^2}{1-\phi^{2n}}\left(z_n - c\frac{1-\phi^n}{1-\phi}\right)^2} \,\mathrm{d}z_n \tag{B.44}$$

$$= \frac{1}{\sqrt{2\pi\sigma^2}} \sqrt{\frac{1-\phi^2}{1-\phi^{2n}}} \underbrace{\int_{y_n - \tilde{h}}^{y_n + \tilde{h}} e^{-\frac{1}{2\sigma^2}\frac{1-\phi^2}{1-\phi^{2n}}\left(z_n - y_n\right)^2} \,\mathrm{d}z_n}_{\geq 2\tilde{h} e^{-\frac{1}{2\sigma^2}\frac{1-\phi^2}{1-\phi^{2n}}\tilde{h}^2}} \tag{B.45}$$

$$\geq \frac{\sqrt{2}\tilde{h}}{\sqrt{\pi\sigma^2}} \sqrt{\frac{1-\phi^2}{1-\phi^{2n}}} e^{-\frac{1}{2\sigma^2}\frac{1-\phi^2}{1-\phi^{2n}}\tilde{h}^2} \tag{B.46}$$

$$\geq \frac{\sqrt{2}\tilde{h}}{\sqrt{\pi\sigma^2}} \sqrt{1-\phi^2} e^{-\tilde{h}^2/(2\sigma^2)}. \tag{B.47}$$

Suppose now $\phi = -1$. We obtain $\mathbb{E}(y_t \,|\, c) = c(1-(-1)^t)/2$ directly from (B.22). By using (B.21) we additionally compute $\mathbb{V}(y_t \,|\, \theta) = t\sigma^2$ which holds for all $t = 1, 2, \ldots$. The result now follows similarly as when $|\phi| > 1$ except that for odd n the maximum wrt. c is obtained when $c = y_n$ and for even n we interestingly have $\mathbb{E}(y_n \,|\, c) = 0$ so that in this case $\mathbb{P}(|z_n - y_n| \leq \tilde{h} \,|\, c)$ is constant wrt. c. In both cases the maximum hence exists and the same upper bound is obtained. For the remaining case $\phi = 1$ we compute $\mathbb{E}(y_t \,|\, c) = tc$ and $\mathbb{V}(y_t \,|\, \theta) = t\sigma^2$ which hold for all $t = 1, 2, \ldots$. The rest again follows similarly as when $|\phi| > 1$.

B.4 Mathematical details related to Example 3.2

We first notice that

$$\begin{bmatrix} y_{1:n} \\ v_{1:n} \end{bmatrix} \Bigg|\, c \sim \mathcal{N}_{2n}\left(c\begin{bmatrix} \mu \\ \mu \end{bmatrix}, \begin{bmatrix} W & \Sigma \\ \Sigma & \Sigma \end{bmatrix} \right), \tag{B.48}$$

where we have $\mu_t = (1-\phi^t)/(1-\phi)$ by (B.22) and where $W = \Sigma + \omega^2 \mathrm{I}$ with $\Sigma_{s,t} = \mathrm{cov}(v_s, v_t \,|\, c)$ by (B.24). Note that μ, Σ and W do not depend on parameter c. We use $\Sigma_{t:}$ to denote the row vector that contains the tth row of Σ and similarly $\Sigma_{:t}$ is the tth column vector. It follows that

$$v_n \,|\, y_{1:n}, c \sim \mathcal{N}(c(\mu_n - \Sigma_{n:}W^{-1}\mu) + \Sigma_{n:}W^{-1}y_{1:n}, \Sigma_{n,n} - \Sigma_{n:}W^{-1}\Sigma_{:n}), \tag{B.49}$$

which is the "batch" solution for the filtering problem. Using $\pi(y_{1:n} \,|\, c) = \mathcal{N}(y_{1:n} \,|\, c\mu, W)$, which follows from (B.48), and some straightforward computations (or, alternatively, by existing formulas for this linear Gaussian inference problem), we see that

$$c \,|\, y_{1:n} \sim \mathcal{N}((\mu^\top W^{-1}\mu)^{-1} \underbrace{\mu^\top W^{-1} y_{1:n}}_{s^{(1)}(y)}, (\mu^\top W^{-1}\mu)^{-1}). \tag{B.50}$$

Now that we have derived (B.49) and (B.50), we can further obtain

$$\pi(y_{n+1} \mid y_{1:n}) = \iiint \mathcal{N}(y_{n+1} \mid v_{n+1}, \omega^2)\mathcal{N}(v_{n+1} \mid c + \phi v_n, \sigma^2)\pi(v_n \mid y_{1:n}, c)\pi(c \mid y_{1:n})\,\mathrm{d}c\,\mathrm{d}v_n\,\mathrm{d}v_{n+1} \tag{B.51}$$

$$= \int \mathcal{N}(y_{n+1} \mid v_{n+1}, \omega^2) \iint \mathcal{N}(v_{n+1} \mid c + \phi v_n, \sigma^2)\pi(v_n \mid y_{1:n}, c)\,\mathrm{d}v_n \pi(c \mid y_{1:n})\,\mathrm{d}c\,\mathrm{d}v_{n+1} \tag{B.52}$$

$$= \mathcal{N}\Big(\phi \underbrace{\Sigma_{n:}W^{-1}y_{1:n}}_{s^{(2)}(y)} + (1 + \phi\mu_n - \phi\Sigma_{n:}W^{-1}\mu)(\mu^\top W^{-1}\mu)^{-1}\underbrace{\mu^\top W^{-1}y_{1:n}}_{s^{(1)}(y)}, \quad \omega^2 + \sigma^2 + \phi^2(\Sigma_{n,n} - \Sigma_{n:}W^{-1}\Sigma_{:n}) + (1 + \phi\mu_n - \phi\Sigma_{n:}W^{-1}\mu)^2(\mu^\top W^{-1}\mu)^{-1}\Big), \tag{B.53}$$

where the integrals in (B.52) are computed in a straightforward manner by using the laws of total expectation and variance.

Finally, we use (B.49) and (B.50) to obtain

$$v_n \mid y_{1:n} \sim \mathcal{N}\Big((\mu^\top W^{-1}\mu)^{-1}\underbrace{\mu^\top W^{-1}y_{1:n}}_{s^{(1)}(y)}(\mu_n - \Sigma_{n:}W^{-1}\mu) + \underbrace{\Sigma_{n:}W^{-1}y_{1:n}}_{s^{(2)}(y)}, \quad \Sigma_{n,n} - \Sigma_{n:}W^{-1}\Sigma_{:n} + (\mu_n - \Sigma_{n:}W^{-1}\mu)^2(\mu^\top W^{-1}\mu)^{-1}\Big). \tag{B.54}$$

The joint Gaussian density of (v_n, c) given $y_{1:n}$ can be formed using (B.54), (B.50) and the fact $\mathrm{cov}(v_n, c \mid y_{1:n}) = (\mu_n - \Sigma_{n:}W^{-1}\mu)(\mu^\top W^{-1}\mu)^{-1}$.

C Additional results

C.1 Additional illustration in the case of Example 3.1

We consider an illustration that is otherwise the same as the one in Example 3.1 except that we use $\phi = 0.99$. The resulting posterior approximations are shown in Figure C.1. The results agree with the theoretical considerations. Interestingly, all summary statistics produce accurate posterior of c in this particular case. The ABC-F posterior predictive densities are not shown for clarity and because they all were essentially the same as the exact posterior. This is not surprising because the ABC posterior for c is accurate in all three cases.

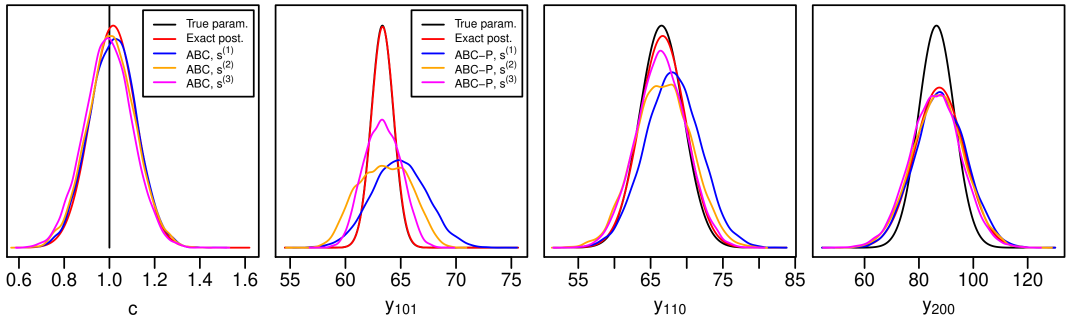

Figure C.1: Illustration of the effect of summary statistics $s^{(1)}(y) = \bar{y}_\phi$, $s^{(2)}(y) = (\bar{y}_\phi, y_n)$ and $s^{(3)}(y) = \mathring{y}_\phi = \bar{y}_\phi + \phi y_n$ on the ABC approximation accuracy. This example is the same as in Figure 1 of the main paper except that here we used $\phi = 0.99$. *The first plot* on the left shows the ABC(-P/F) posterior for c and *the three other plots* the ABC-P posterior predictive distribution at some future time points.

C.2 Computed ABC posteriors for the missing data case of Section 5.2.3

Finally, Figure C.2 shows the computed ABC posterior distributions for the parameters of the Lotka-Volterra experiments of Section 5.2.3. We can see that both data realizations lead to fairly similar ABC posteriors in this case.

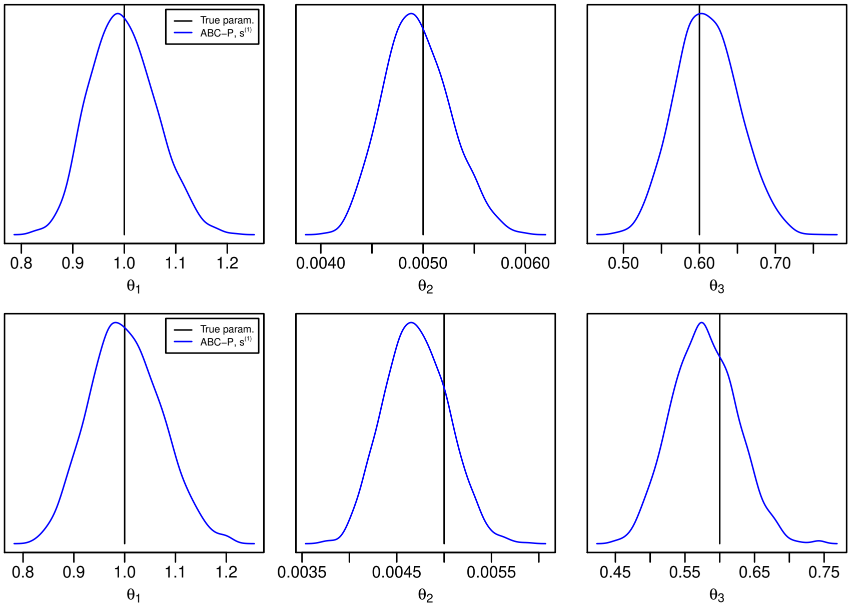

Figure C.2: Posterior distributions for the parameters of the Lotka-Volterra experiments corresponding to Figure 7 in Section 5.2.3 of the main paper. *Top row:* The first realization of data. *Bottom row:* The second realization of data where the populations have become extinct. The black vertical line shows the true value of the parameter.

References

Rudin, W. (1987). *Real and complex analysis.* McGraw-Hill, third edition.

Stein, E. M. and Shakarchi, R. (2005). *Real Analysis: Measure Theory, Integration, and Hilbert Spaces.* Princeton University Press.
